# Constructing and interpreting a large-scale variant effect map for an ultrarare disease gene: Comprehensive prediction of the functional impact of *PSAT1* genotypes

**DOI:** 10.1371/journal.pgen.1010972

**Published:** 2023-10-09

**Authors:** Michael J. Xie, Gareth A. Cromie, Katherine Owens, Martin S. Timour, Michelle Tang, J. Nathan Kutz, Ayman W. El-Hattab, Richard N. McLaughlin, Aimée M. Dudley

**Affiliations:** 1 Pacific Northwest Research Institute, Seattle, Washington, United States of America; 2 Molecular Engineering Graduate Program, University of Washington, Seattle, Washington, United States of America; 3 Department of Applied Mathematics, University of Washington, Seattle, Washington, United States of America; 4 Department of Clinical Sciences, College of Medicine, University of Sharjah, Sharjah, United Arab Emirates; University of Toronto, CANADA

## Abstract

Reduced activity of the enzymes encoded by *PHGDH*, *PSAT1*, and *PSPH* causes a set of ultrarare, autosomal recessive diseases known as serine biosynthesis defects. These diseases present in a broad phenotypic spectrum: at the severe end is Neu–Laxova syndrome, in the intermediate range are infantile serine biosynthesis defects with severe neurological manifestations and growth deficiency, and at the mild end is childhood disease with intellectual disability. However, L-serine supplementation, especially if started early, can ameliorate and in some cases even prevent symptoms. Therefore, knowledge of pathogenic variants can improve clinical outcomes. Here, we use a yeast-based assay to individually measure the functional impact of 1,914 SNV-accessible amino acid substitutions in PSAT. Results of our assay agree well with clinical interpretations and protein structure-function relationships, supporting the inclusion of our data as functional evidence as part of the ACMG variant interpretation guidelines. We use existing ClinVar variants, disease alleles reported in the literature and variants present as homozygotes in the primAD database to define assay ranges that could aid clinical variant interpretation for up to 98% of the tested variants. In addition to measuring the functional impact of individual variants in yeast haploid cells, we also assay pairwise combinations of *PSAT1* alleles that recapitulate human genotypes, including compound heterozygotes, in yeast diploids. Results from our diploid assay successfully distinguish the genotypes of affected individuals from those of healthy carriers and agree well with disease severity. Finally, we present a linear model that uses individual allele measurements to predict the biallelic function of ~1.8 million allele combinations corresponding to potential human genotypes. Taken together, our work provides an example of how large-scale functional assays in model systems can be powerfully applied to the study of ultrarare diseases.

## Author summary

Serine metabolism is central to numerous biological processes, including the synthesis of proteins, nucleotides, and phospholipids, and the neuromodulators D-serine and glycine. Genetic variants that impair the function of one of the human enzymes involved in serine biosynthesis, PSAT, result in inherited metabolic diseases with a wide array of phenotypes, ranging from childhood disease with intellectual disability to the severe Neu-Laxova syndrome. Knowing which variants impair PSAT function, to a degree sufficient to cause disease, is important for early diagnosis and treatment of these disorders. Currently, this information is available for only a very small number of variants. Here, we implement a high throughput assay using haploid yeast cells to individually measure the effect of 1,914 amino acid substitutions on human PSAT function. Using a small number of clinically annotated variants, we define assay ranges that could aid clinical interpretation for up to 98% of the full set of tested variants. We also construct and assay yeast diploids with pairwise combinations of *PSAT1* alleles that model human genotypes.

## Introduction

Inborn errors of metabolism (IEMs), genetic alterations that disrupt metabolic processes, underlie a relatively large number of rare diseases. Over 700 have been described in the literature, and new disorders continue to be discovered [1,2]. Although individually rare, collectively these diseases are common, with estimates suggesting that IEMs may affect 1 in 800–2600 live births [3,4]. Because disruptions of metabolic pathways can lead to toxic levels of substrate accumulation or deficiency of an essential product, many IEMs are severe and manifest early in life. Therapeutic strategies often focus on amending these imbalances. For example, dietary restriction is prescribed for the treatment of urea cycle disorders, phenylketonuria, and galactosemia, while dietary supplementation is prescribed for homocystinuria and pyridoxine-dependent epilepsy [5–7]. For many of these diseases, timely diagnosis and therapeutic intervention can prevent the onset of irreversible damage.

Serine biosynthesis defects are ultrarare diseases caused by impairment of any of the three L-serine biosynthesis pathway enzymes, phosphoglycerate dehydrogenase (PGDH; encoded by *PHGDH* [MIM: 606879]), phosphoserine aminotransferase (PSAT; encoded by *PSAT1* [MIM: 610936]), and phosphoserine phosphatase (PSP; encoded by *PSPH* [MIM: 172480]) [8]. Serine metabolism is central to numerous biological processes, including the synthesis of proteins, nucleotides, and phospholipids, as well as the formation of the neuromodulators D-serine and glycine. As a result, serine biosynthesis defects negatively impact nervous system development and function, with clinical manifestations that include microcephaly, seizures, intellectual disability, and neuropathies. Case studies have demonstrated that oral serine supplementation can reduce and, in some cases, prevent the onset of these severe symptoms that typically manifest very early in life (S1 Fig) [9–13]. Prompt diagnosis is crucial, as the impact of therapeutic intervention, including prenatal maternal dietary supplementation, is greatest before individuals become symptomatic and irreversible neurological damage occurs.

One challenge in diagnosing serine deficiency disorders is their broad phenotypic spectrum [8,14]. At one extreme, Neu-Laxova syndrome (NLS) is a severe, lethal, multiple congenital anomaly disease characterized by marked intrauterine growth restriction, microcephaly, distinctive facial features, limb and skin defects, and variable brain malformations including lissencephaly, corpus callosum agenesis, and hypoplastic cerebellum and pons. Infantile serine biosynthesis defects represent the intermediate form of the disorder spectrum, typically presenting with intrauterine growth restriction, microcephaly, feeding difficulties, vomiting, irritability, hypertonia evolving into spastic tetraplegia, nystagmus, early onset seizures, and poor psychomotor development. Other findings include congenital cataracts, adducted thumbs, megaloblastic anemia, brain atrophy, hypomyelination, and hypoplastic cerebellum and pons. At the mild end of the disorder spectrum are childhood serine biosynthesis defects characterized by developmental delay, intellectual disability, ichthyosis [15], and behavioral abnormalities. Other findings include epilepsy, ataxia, nystagmus, polyneuropathy, and hypertonia. Although it has been hypothesized that residual enzyme activity may explain the phenotypic variability [16–18] within serine deficiency disorders, functional studies regarding this subject are sparse and the natural histories of these diseases are largely unknown.

Biochemical diagnosis of serine biosynthesis defects is most accurately performed by the measurement of amino acid levels in serum or cerebral spinal fluid (CSF), with comparison to closely age-matched controls. Although the serum-directed metabolic screen is more widely used and is considered generally in children with intellectual disability, serine and glycine plasma concentrations can be normal in the presence of serine biosynthesis defects if the blood sample is not obtained in a fasted state. In contrast, CSF serine and glycine concentrations are not affected by meals, however, CSF amino acid analysis is not typically considered in the absence of seizures. Therefore, the metabolic work up may fail to identify children with serine biosynthesis defects [19,20].

Whole exome or genome sequencing has the potential to improve diagnosis of inherited metabolic diseases, such as serine biosynthesis defects, shortening a patient’s diagnostic odyssey and speeding time to treatment. Unfortunately, clinical interpretations for observed variants are generally not available, particularly for rare and ultrarare variants. Most of the ~550K missense variants in ClinVar [21], even those in well-studied disease genes, are variants of uncertain significance (VUS). The American College of Medical Genetics (ACMG) has developed a series of guidelines for genetic variant interpretation [22]. This framework weights evidence from multiple types of data (e.g population frequency, genetic segregation, functional assays, and computational predictions) to make assertions towards pathogenic or benign variant interpretations. Although computational prediction methods can be applied at scale, their relatively high error rates limit their utility [23]. Functional data can potentially contribute very strong levels of evidence (e.g PS3 or BS3) within the criteria considered, but is rarely available, and until recently has been generated using relatively low throughput methods. However, improvements in DNA synthesis technology have enabled time-and-cost effective methods for building large variant libraries. When combined with high-throughput phenotyping methods, this allows functional assays to comprehensively assess the functional impact of variants that have been identified in the human population as well as those that may arise in the future [24,25].

Recently, our laboratory established a yeast-based functional assay for human PSAT [26], an enzyme that catalyzes the reversible conversion of glutamate to α-ketoglutarate and 3-phosphohydroxypyruvate (3-PHP) to phosphoserine [27]. Here, we apply it at scale to quantify the functional impact of 1,914 amino acid substitutions that can be introduced via a single nucleotide variant (SNV-accessible) in the human *PSAT1* coding sequence. Results of our assay agree well with clinical interpretations and protein structure-function relationships, supporting the use of our data as functional evidence, which may be used to help classify variants under the ACMG interpretation guidelines. In addition to assaying the functional impact of single variants in yeast haploids, we construct and assay yeast diploids with pairwise combinations of *PSAT1* alleles that recapitulate human genotypes. The results of this diploid functional assay show a clear separation between the scores of patient genotypes and those of healthy carriers. Our results also show quantitative agreement between the degree of functional impairment in our assay and patient disease severity. Finally, we develop a mathematical model that can accurately predict biallelic function in diploids from pairwise combinations of individual allele activities measured experimentally in haploids. Taken together, our work provides an example of how large-scale functional assays in model systems can be powerfully applied in the study of ultrarare disease.

## Results

### A large-scale survey of the functional impact of missense variation in *PSAT1*

Our laboratory had previously established a yeast-based complementation assay that leveraged the ability of the human *PSAT1* coding sequence to functionally replace its yeast ortholog, *SER1* [26]. In this assay, growth on minimal medium lacking serine provides a quantitative readout of PSAT activity, allowing the functional impact of protein coding variants to be assessed. Variant impact is expressed on a relatively intuitive scale of activity between zero and one, with zero representing the level of yeast growth associated with no activity (that of a complete gene deletion), and one representing the level conferred by wild type human PSAT. In this assay, reduced PSAT function could result from decreases in enzymatic activity, altered protein stability, or a combination of both.

**Fig 1:**
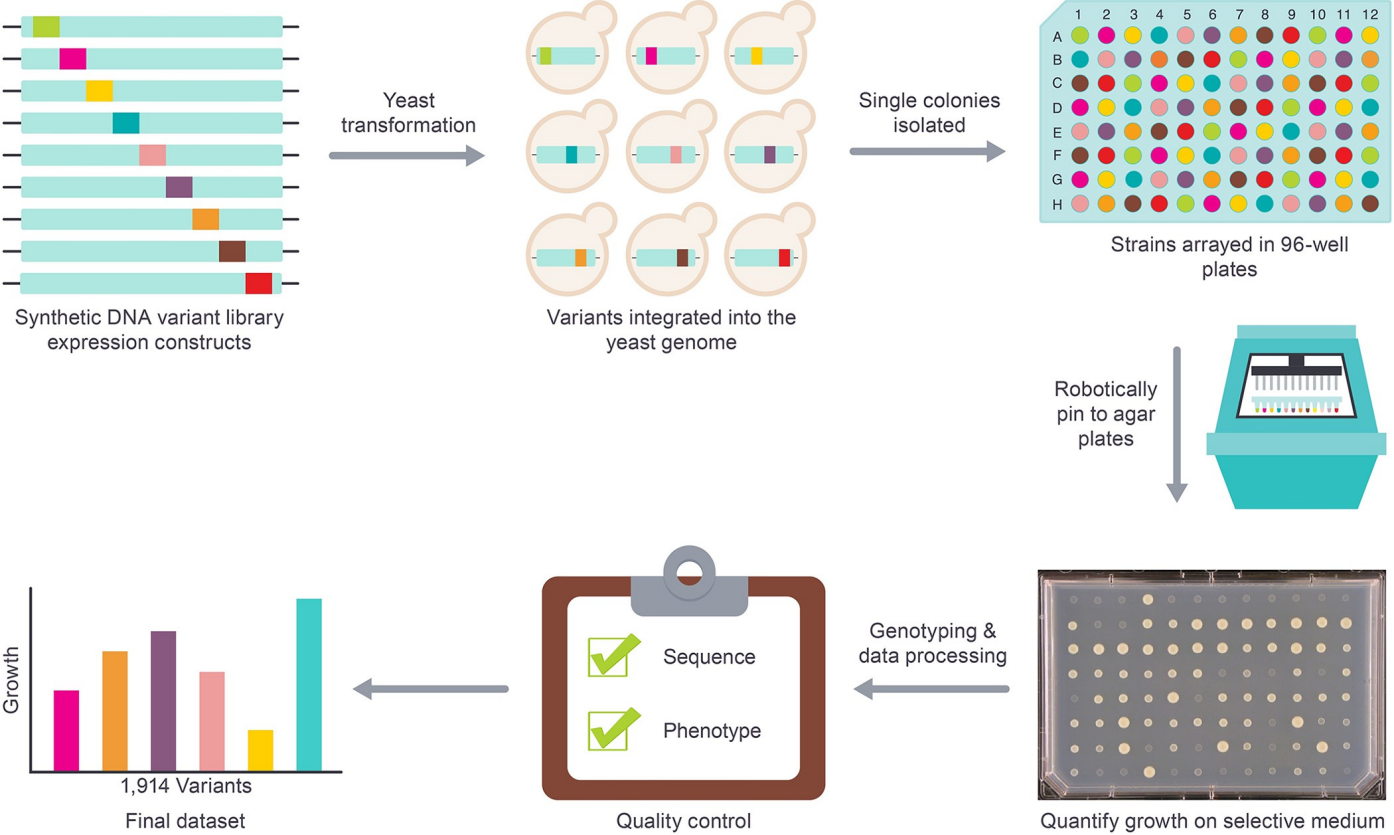
High-throughput measurement of PSAT function. A synthesized DNA library of *yPSAT1* variants is transformed into the genome of the yeast deletion strain (*ser1Δ0*) and arrayed into 96-well plates. Strains are robotically pinned (in triplicate) to medium lacking serine, growth is measured by image analysis, and normalized relative to the growth of yeast cells harboring the wild type *yPSAT1* and strains lacking PSAT activity (*ser1Δ0*). Following strain genotyping, data processing, and several quality control steps, the final dataset provides functional impact estimates for 1,914 PSAT variants.

Here, we applied the assay at scale to measure the effect of thousands of amino acid substitutions as follows (Fig 1). First, with the exception of the translational initiation and termination codons, we identified all unique amino acid substitutions (n = 2,182) that were accessible via a SNV across the full length of human *PSAT1* isoform 1 cDNA (1,113 bp). A single variant codon for each amino acid substitution was then introduced into the yeast codon-optimized version of *PSAT1* (*yPSAT1*). The resulting variant library was transformed into a haploid *SER1* deletion strain (*ser1Δ0*) and integrated in single copy at the *SER1* locus of the yeast genome, under the control of the endogenous *SER1* transcriptional promoter and terminator. Next, transformants were individually arrayed in 96-well plate format, and the identity of the variant codon present in each transformant was determined by Oxford Nanopore MinION sequencing (Methods). Strains that harbored secondary mutations in the protein coding sequence were removed from further consideration. The arrayed strain library was then grown, in triplicate, on solid medium lacking serine and imaged after three days at 30°C. Data from these images were used to quantify the growth of each strain, relative to wild type (*yPSAT1*) and null (*ser1Δ0*), using a custom automated image analysis pipeline and normalization software (Methods).

After applying genotyping and phenotyping quality filters (Methods), our final dataset consisted of functional impact estimates for 1,914 amino acid substitutions in *PSAT1* (S3 Table), corresponding to ~88% of all unique SNV-accessible amino acid substitutions (n = 2,182) in the human *PSAT1* cDNA sequence. A subset of these substitutions (n = 196) had also been assayed in our previous study [26], which included the full set of *PSAT1* missense variants described in ClinVar, gnomAD [28], or the clinical literature at that time. Reanalysis in the current study allowed us to assess these amino acid substitutions using the same improved data processing pipeline as the 1,718 new substitutions, thereby placing them on a common scale and facilitating direct comparison. Despite the use of slightly different data analysis pipelines, there was very close agreement (R^2^ = 0.94) between the normalized growth estimates of the 196 substitutions in the two studies.

**Fig 2:**
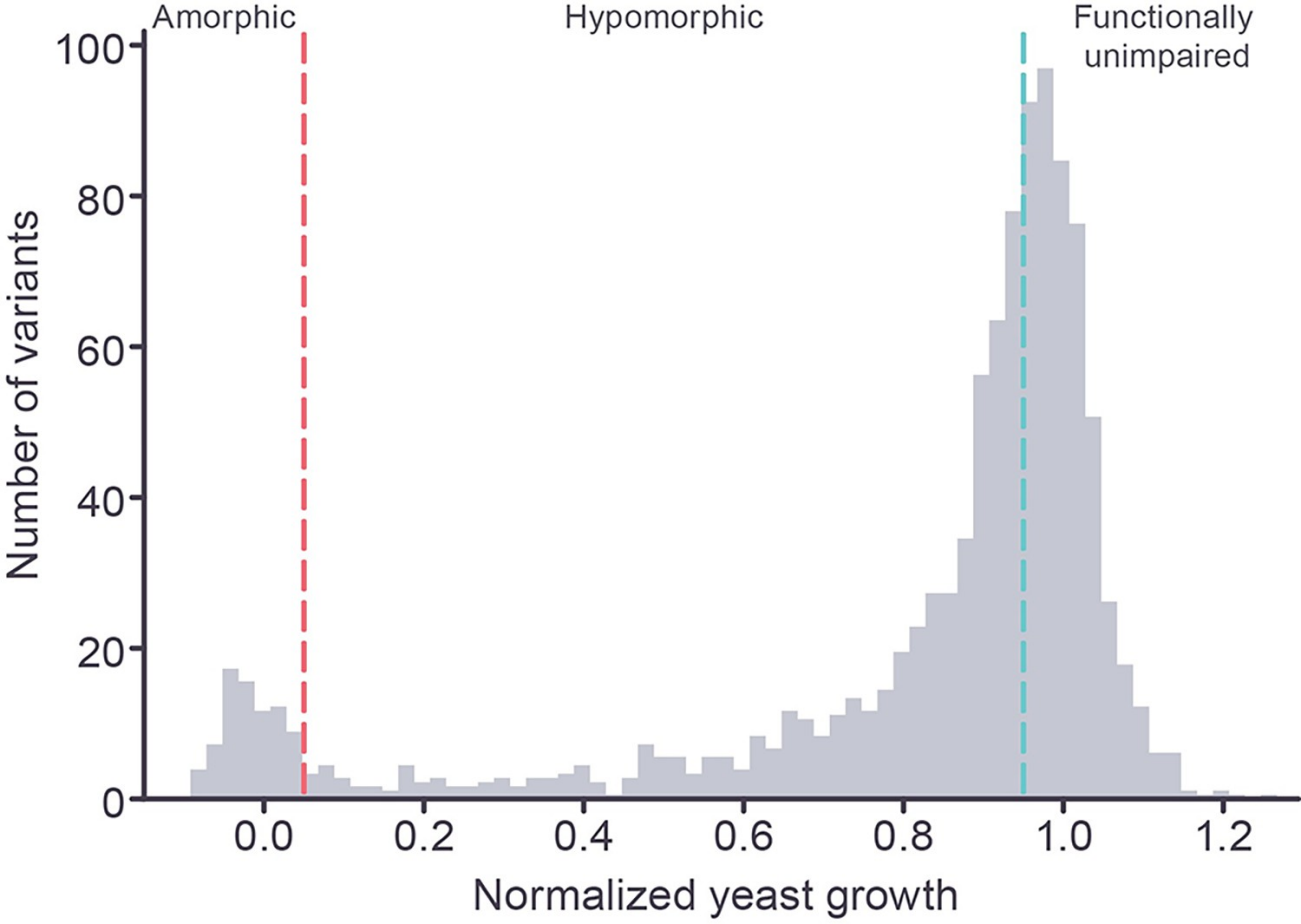
Distribution of variant effects for 1,914 PSAT1 unique SNV-accessible amino acid substitutions. The frequencies (in 2% intervals) of experimentally measured yeast growth values scaled relative to wild type *yPSAT1* (normalized growth = 1) and null (normalized growth = 0) are shown. The amorphic, hypomorphic, and functionally unimpaired growth ranges are delineated by the red (0.05) and blue (0.95) dashed lines.

Among the full set of 1,914 amino acid substitutions, the distribution of growth values was bimodal, with a large peak centered near the value of the wild type *yPSAT1* strain (normalized growth = 1) and a smaller peak centered around the value of the null (deletion) strain (normalized growth = 0) (Fig 2). These results are consistent with a large group of protein coding variants showing little or no functional impairment relative to wild type, and a smaller group behaving as complete loss of function alleles. The remaining protein coding variants showed varying levels of functional impairment (Fig 2). The approximate bimodal distribution of our dataset, with modes centered on null and wild type scores, is similar to functional scores generated by deep mutational scanning studies of other proteins assayed in human cell lines (BRCA1 [29], PTEN [30], TPMT [30], and VKOR [31]), and in yeast cells (MTHFR [32], CYP2C9 [33],CBS [34]).

On the basis of the observed distribution of growth scores, we classified amino acid substitutions in our assay as follows. Substitutions with ≤95% normalized growth were considered functionally impaired relative to wild type and the remaining substitutions (>95% normalized growth) were considered unimpaired. Among the impaired class, we further defined any substitutions resulting in ≤5% normalized growth (i.e. comparable to that of the null control) as amorphs, and any substitutions resulting in less severe functional impairment (>5% and ≤95% normalized growth) as hypomorphs.

### Structural insights from PSAT functional scores

Variant effect mapping is a powerful tool for investigating protein structure-function relationships. However, the limited size of the dataset in our previous study [26], precluded this kind of analysis. The current study makes 4–7 amino acid substitutions at each position across the length of the protein, and therefore constitutes a much more densely sampled set of amino acid substitutions. Thus, we utilized our variant data to investigate structure-function relationships for PSAT. We investigated how well the results from our functional assay agreed with known structural features of the protein, and also the extent to which our dataset could be used to provide a better understanding of regions of the protein that are not as well characterized.

**Fig 3:**
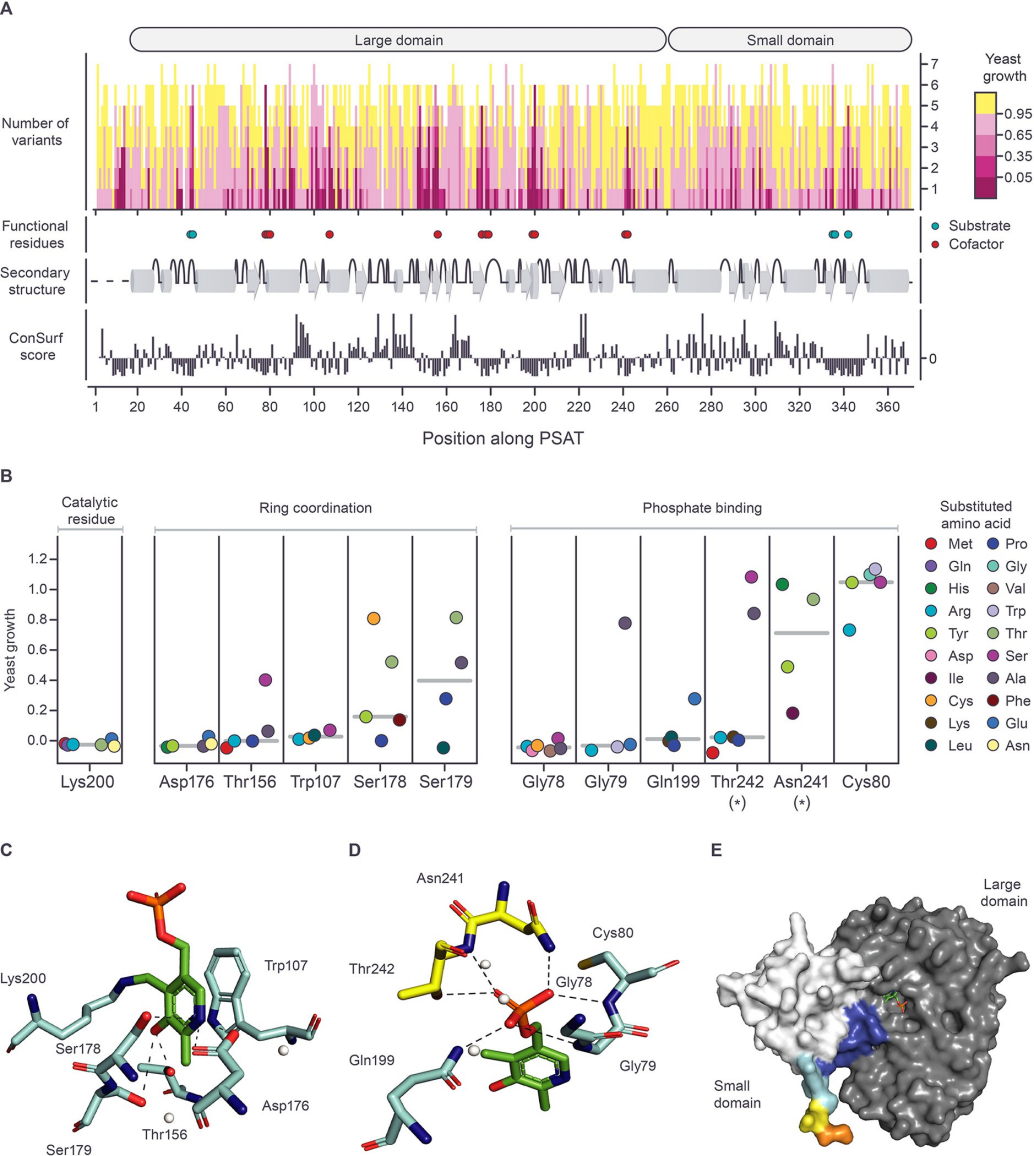
Missense variant effect map across the length of human PSAT compared to structural features. **(A)** Growth values for the substitutions introduced at each amino acid position, ordered from highest (top) to lowest (bottom) growth. Positions of the functional residues of PSAT, secondary structure, and ConSurf evolutionary conservation scores are indicated below. Subunit domains are indicated above. Residues implicated in substrate binding or cofactor binding are shown as circles (righthand legend). Helices and beta-sheets are depicted as gray cylinders and arrows, respectively. Turns shown as upward half-coils. The black dotted line represents secondary structures that were unavailable due to absence from the solved crystal structure (PDB: 3e77; Leu17-Leu370). More negative ConSurf scores indicate more conserved positions. **(B)** The distribution of variant growth scores for cofactor binding residues. Each circle is colored according to the amino acid that is substituted, as shown in the legend. Asterisks denote amino acids from the other subunit. The median growth score for each position is shown as a horizontal grey bar. **(C-D)** Organization of PLP cofactor binding residues. Stick representations for residues and the PLP cofactor are colored by element (carbon: cyan for subunit one, carbon: yellow for the opposite subunit, carbon: green for PLP, nitrogen: blue, oxygen: red, phosphate: orange). Hydrogen bonding is depicted by dashed black lines and nearby water molecules are represented by small white spheres. **(C)** Residues that coordinate PLP’s heterocyclic ring, with the catalytic lysine shown covalently bound to PLP. **(D)** Residues that bind to the phosphate group of PLP. **(E)** Surface representation of the full-length subunit structure for human PSAT, as predicted by AlphaFold. N-terminal residues (Met1-Lys16) are colored by the standard AlphFold pLDDT confidence measure, with very confident (pLDDT >90) scores shown as dark blue, confident scores (90 > pLDDT > 70) as light blue, low scores (70 > pLDDT > 50) as yellow, and very low (pLDDT <50) scores as orange. The remaining residues are colored by their subunit domain in grey and white. The full predicted structure colored by the default AlphaFold color scheme is available in S4 Fig. The PLP cofactor (green) is represented as a stick and was transplanted into the predicted structure using AlphaFill, to indicate the location of one of the active sites.

We first mapped the distribution of growth scores per residue onto the linear sequence of the protein (Fig 3A). As expected, we observed varied sensitivity to amino acid substitution at different residues of the protein. At some positions, the majority of SNV-accessible amino acid substitutions exhibited some degree of functional impairment, while other positions were mutationally tolerant to all variants tested (Fig 3A). We then compared these patterns to known features of the protein structure, such as the cofactor and substrate binding sites. Since PSAT is a homodimer (S2 Fig) that requires pyridoxal phosphate (PLP; the active form of vitamin b6) for catalysis, we expected residues involved in coordination of this cofactor would be sensitive to substitution [35]. From the crystal structure of human PSAT complexed with PLP (PDB: 3e77, residues Leu17-Leu370;), we identified 12 residues that directly interact with the functional groups of the cofactor at the active sites (Fig 3C and 3D). Consistent with expectation, we observed that the median growth of variants at these positions was only 7.3%, a value significantly lower than the global median of 93.6% (p< 1x10^-5^, one sided permutation test). Thus, residues of human PSAT with established roles in cofactor coordination exhibit the expected sensitivity to amino acid substitution in the yeast system.

We next examined the functional impact of variants at each of these cofactor-interacting positions individually. We expected that residues involved in coordination of the pyridoxal ring group (Fig 3C) would be sensitive to substitution, as this functional group is directly involved in catalysis [35–37]. In fact, all tested substitutions at these six residues (Lys200, Asp176, Thr156, Trp107, Ser178, and Ser179) were functionally impaired (Fig 3B). The majority of these substitutions (19/29), including all SNV-accessible missense variants at the catalytic lysine (Lys200), were amorphic in our assay (Fig 3B). Next, we examined the six residues that participate in phosphate group binding (Fig 3D), whose functional role is less well understood. Other studies have suggested that the phosphate group may act as an anchor point to the protein [38], or even directly interact with nearby substrates [37]. At three of these positions (Gly78, Gly79, and Gln199) all variants were functionally impaired and had median growth scores in the amorphic range (Fig 3B). Functionally impaired variants were also observed at Asn241, and Thr242 although some substitutions at these sites displayed unimpaired growth (Fig 3B). Interestingly, with the exception of p.Cys80Arg (growth score = 80%), all substitutions at the Cys80 residue were not impaired relative to wild type. It has been hypothesized that Cys80 contributes to the higher phosphoserine substrate affinity (Km = 5 μM) observed in human PSAT relative to other phosphoserine aminotransferases [39], and possibly the positive charge introduction represented by p.Cys80Arg interferes with binding negatively charged substrates.

Next, we examined residues of PSAT that are likely important for substrate interaction. Because the human PSAT crystal structure was solved without bound substrate, the interaction between the substrate and specific residues in the active site can only be inferred. We therefore considered our functional results in the context of the substrate-bound crystal structures of three orthologous phosphoserine aminotransferases from *Escherichia coli* [40], *Bacillus alcalophilus* [41], and *Arabidopsis thaliana* [42]. These structures identified five putative substrate binding residues. Consistent with these residues having an important role in the function of the enzyme, amino acid substitutions at four of these sites (His335, Arg342, His44 and Arg45) in human PSAT, showed strong loss of function (S3 Fig). The remaining site (Arg336) displayed activity similar to wild type for all tested substitutions. Thus, Arg336 may be a conserved residue that participates in substrate stabilization in other organisms, but not in humans.

In addition to capturing known structural features, our dataset also provides insight into less well characterized regions of the protein. The solved human PSAT crystal structure lacks the first 16 N-terminal amino acids (PDB: 3e77), in which we observe a cluster of sites intolerant to amino acid substitution (residues Gly11-Pro14) (Fig 3A). AlphaFold [43,44] predicts that several of these residues (Val7-Lys16) are located in the small domain of the protein, proximal to the active site (Figs 3E and S4). Interestingly, a deletion of four N-terminal residues in *Entamoeba histolytica* phosphoserine aminotransferase, corresponding to Pro12-Ala15 in human PSAT, yielded a mutant enzyme that had reduced substrate (phosphoserine) affinity, as well as a 10-fold reduction in activity [45]. Additionally, a serine residue involved in analog substrate binding has been identified in *E*. *coli* [40], corresponding to Pro12 in human PSAT. Thus, functional information from our assay, together with structural predictions, suggests that a previously uncharacterized region of the human protein may be important for substrate binding.

### Amino acid substitutions are frequently tolerated at conserved PSAT residues

In addition to mechanistic insights, our large, densely sampled dataset can also be used to address broader questions relating to the conservation of the protein. To quantify the relative conservation of residues in human PSAT, we used ConSurf [46], which measures conservation as the evolutionary rate observed at each position within a multiple alignment of orthologs. We used the ConSurf server to compute conservation scores for PSAT derived from a multiple sequence alignment that includes other phosphoserine aminotransferases from eukaryotic and prokaryotic organisms. Negative ConSurf scores represent more conserved amino acid positions, and positive scores represent less conserved residues.

**Fig 4:**
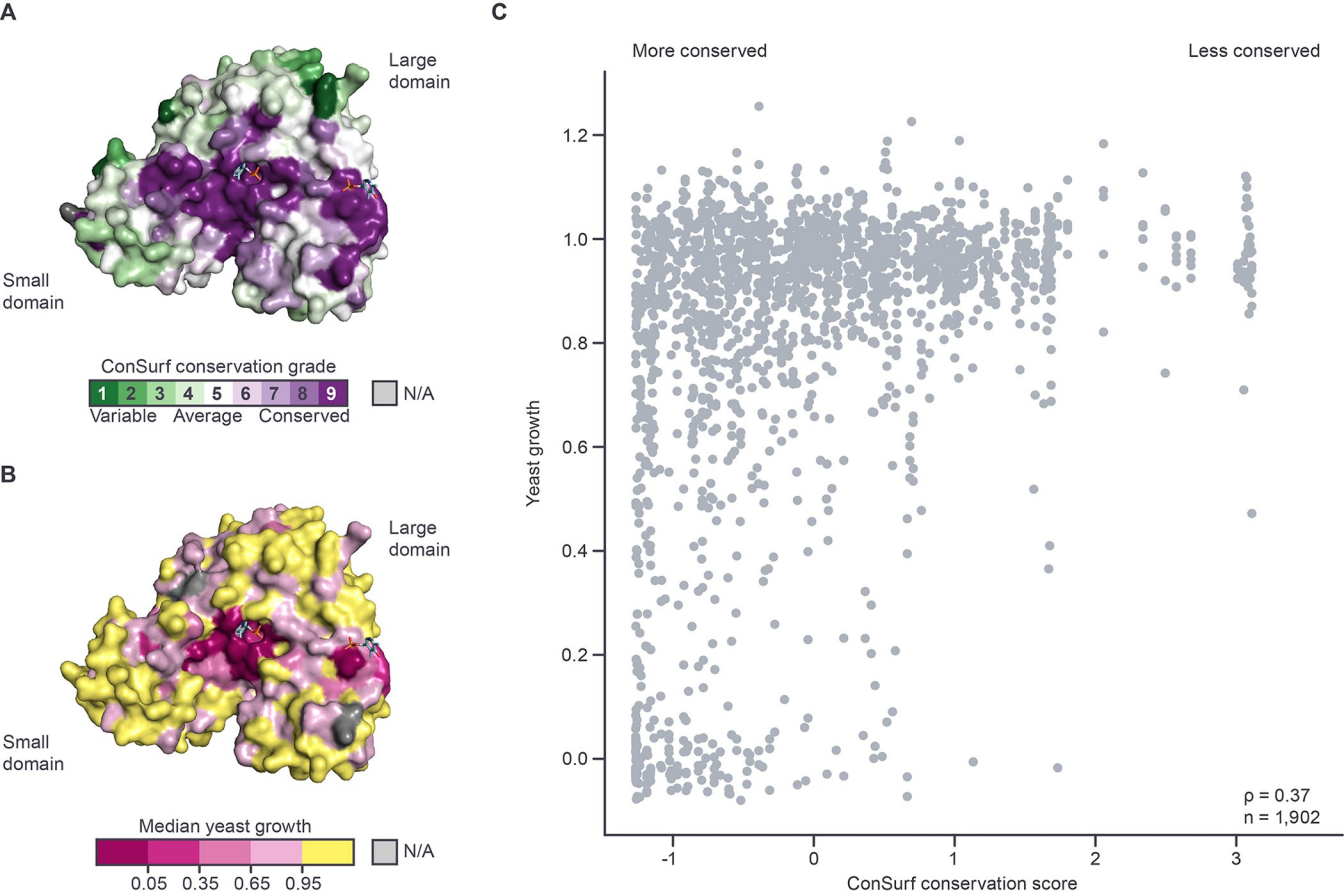
PSAT functional scores compared to evolutionary conservation. **(A-B**) PSAT subunit surface representations (PDB: 3e77, Leu17-Leu370) colored by the ConSurf evolutionary conservation grade **(A)** and median yeast growth estimate **(B)** per residue. Two PLP cofactors (cyan) are shown as sticks to indicate the location of the two active sites. **(C)** Scatterplot of yeast growth scores versus ConSurf conservation scores for all amino acid substitutions tested. Each circle represents a single variant. Total number of variants and Spearman rank correlation labeled. More (< 0) or less (> 0) conserved ConSurf scores are labeled above.

Because phosphoserine amino transferases belong to the class IV family of aminotransferases, which share a similar overall structural organization and sequence motifs at the active site [35], we expected that highly conserved residues in PSAT would be sensitive to amino acid substitutions. Consistent with this hypothesis, there was a moderately strong, but highly significant, correlation (Spearman rank correlation = 0.47, p < 7.2 x 10^−22^, S5 Fig) between the ConSurf score for a residue and the median yeast assay score for substitutions at that site, with more conserved residues displaying lower median growth. Mapping ConSurf scores and median yeast growth onto the PSAT crystal structure (Fig 4A and 4B) revealed the following pattern. Immediately adjacent to the cofactor binding site, residues are highly conserved and sensitive (low median growth scores) to amino acid substitution. Further from the active site, there is a region consisting of residues that are also highly conserved, but which are tolerant (high median growth scores) to substitution. The remaining residues, furthest from the active site, are generally poorly conserved and tolerant to amino acid substitution.

To further explore the observation that more conserved residues tend to be more sensitive to amino acid substitutions, we next examined the strength of the relationship between the functional effect of individual amino acid substitutions and the degree of conservation of the residue at which they occur. Consistent with expectation, substitutions with amorphic growth in our assay were concentrated at more highly conserved residues (Fig 4C), with 92% of amorphic variants having ConSurf scores <0, versus 55% of all variants (p = 9.6 x 10–23; one-sided Fisher’s Exact test). However, many amino acid substitutions at conserved residues showed little effect on PSAT function in our assay. Among the 1,051 tested substitutions at more conserved amino acid positions (ConSurf score < 0), 127 were amorphic, 574 were hypomorphic, and 350 were unimpaired. Thus, although the majority of amino acids changed at conserved residues had a significant impact on PSAT function, a substantial fraction (33%) did not.

The propensity of conserved alleles to tolerate many amino acid substitutions is a known phenomenon that confounds *in silico* predictive tools that utilize evolutionary conservation, either as their primary basis or in combination with other features. These predictions often overestimate the effects of missense substitutions as deleterious [47]. We therefore compared our growth scores to several widely used computational predictors and observed a moderate positive correlation (mean absolute Spearman rank correlation = 0.45, S6 Fig). However, consistent with the observation that most variant effect predictors suffer from low specificity, we found that many predicted scores overestimated deleterious effects relative to our assay (S6 Fig).

### Mapping functional effects to clinical interpretations

While not sufficient alone to classify variants, data from validated functional assays are valuable as one of several potential lines of supporting evidence for variant annotation according to the ACMG guidelines [22]. Functional assays allow the quantification of impairment of protein activity with a certain level of sensitivity. One challenge in using functional data to inform variant interpretation is understanding the relationship between activity measured in a functional assay and its likely clinical impact. At the simplest level, when reduced protein activity is expected to lead to pathogenicity, it should be possible to define a threshold on the scale of the functional assay below which a disease state results. However, as this value is not known *a priori*, variants with existing clinical annotation must be used to identify a threshold within the assay below which variants are expected to be pathogenic and above which they are expected to be benign.

**Fig 5:**
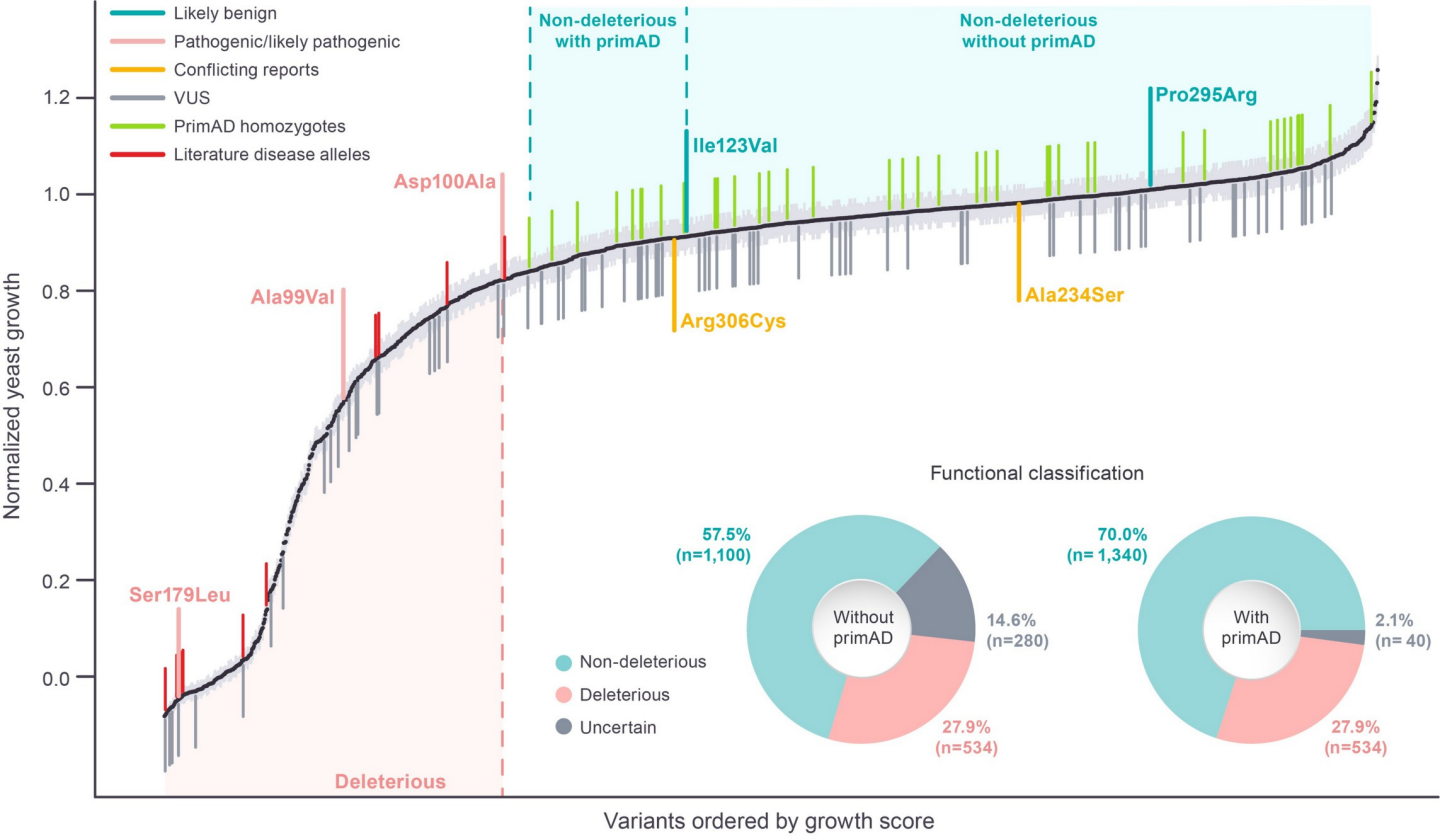
Mutational scan of 1,914 PSAT1 unique SNV-accessible missense amino acid substitutions. Rank ordered (lowest to highest) normalized variant growth. Black dots represent the mean normalized growth estimate for each substitution, with light grey bars indicating standard errors. Colored lines extending above and below each growth score illustrate whether that variant is currently annotated in ClinVar, the disease literature, or primAD (available in S3 Table). The labeled disease alleles represent missense alleles reported in the literature that do not have pathogenic/likely pathogenic classification in ClinVar. primAD labeled variants are those that have been observed in non-human primate homozygotes. The stratification of normalized growth estimates, based on thresholds derived from ClinVar clinical annotation with and without primAD data, into functional classifications are shown by dotted lines with shaded ranges. The subpanel pie charts show the distribution of functional classifications for each thresholding approach applied to the entire score set.

On this basis, we began by benchmarking our assay ranges using the scores of the small number of amino acid substitutions with definitive clinical significance calls (Figs 5 and S7). We first identified a deleterious (supporting pathogenicity) range of the assay (≤82% growth), below which reside all three of the substitutions derived from known pathogenic variants (Figs 5 and S7 and S8). We next identified a non-deleterious (supporting benignity) range (≥91% growth), above which reside substitutions derived from the two known benign variants (Figs 5 and S7A). Notably, the distribution of benign alleles, and the associated cutoff of 91% growth, suggest that variants causing small reductions in PSAT function (hypomorphs with 91–95% growth) are not deleterious/pathogenic. Because increases in enzyme activity above wild type have not been associated with serine deficiency disorders, we included values greater than wild type in the non-deleterious range. Finally, because there are no clinically annotated variants associated with substitutions having scores between 82% and 91%, this range of the assay is of unknown clinical significance, and this range of the functional assay would be considered uncertain.

Ideally, formal measures of classification confidence, such as odds of pathogenicity, would be assigned to these assay ranges [48]. A challenge for rare diseases, such as serine deficiency disorders, is that the limited number of well characterized pathogenic and benign variants precludes the use of such approaches. At the time of writing, for *PSAT1* there are only three missense variants listed in ClinVar with pathogenic/likely-pathogenic annotation, and only two annotated as benign/likely-benign (Figs 5 and S7). As with our previous study [26] these limited numbers precluded us from assigning confidence estimates to our thresholding-based classifications.

After applying the classification thresholds to our variant growth data, 534 (28%) of all amino acid substitutions tested are defined as deleterious, 1,100 (57%) are non-deleterious, and 280 (15%) are uncertain (Fig 5). Among the variants that we tested, 11 have been described in the disease literature (S4 Table) but have not been assigned pathogenic/likely-pathogenic classifications in ClinVar. Supporting the validity of our assay classification ranges, all but one of these variants fall in the deleterious range of our assay, with the final variant falling just above the deleterious boundary, in the uncertain range. In addition, 80 ClinVar missense variants currently annotated as VUS were tested in our assay. Of these, 22 result in substitutions that fall in our deleterious range, 41 in our non-deleterious range and 17 in the uncertain range. Thus, our dataset provides functional information for a large number of variants that lack clinical interpretation, including deleterious or non-deleterious assignments for 77% of the missense VUS currently listed in ClinVar.

We next examined our data in the context of allele frequencies in the human population using the gnomAD [28] database (S9 Fig). Among the variants tested in our assay, 195 were present in gnomAD (v2.1.1). The sole likely pathogenic variant p.Ala99Val present in gnomAD is a known disease variant whose relatively high frequency is driven by a known founder effect in populations of Middle Eastern origin [18]. Consistent with its pathogenicity, this allele has a significant growth defect in our assay (growth score = 57%; S9 Fig) and is not reported in gnomAD in the homozygous state. The four variants with the highest allele frequencies in gnomAD include two likely benign variants (p.Ile123Val and p.Pro295Arg) and two variants (p.Arg306Cys and p.Ala234Ser) with conflicting reports of pathogenicity (S9 Fig). Each of these conflicting reports includes one report specifying benign or likely benign and another specifying VUS. All four of these high growth variants have activities similar to wild type, and two (p.Ala234Ser and p.Ile123Val) are the only *PSAT1* missense variants that are found as homozygotes in gnomAD. Thus, the results of our assay lend additional support to established benign variants and support benign interpretations for p.Ala234Ser and p.Ile123Val.

A recent publication [49] and database (primAD, https://primad.basespace.illumina.com/), described variants in human proteins that are also observed in other primates. Because of the short evolutionary distance between humans and other primates, and the small number of differences between primate protein coding sequences, it is expected that variants impairing protein function in one primate species should also impair function in other species. For genes associated with severe developmental disease, such as *PSAT1*, such variants should be under purifying selection in all species, so that variants observed in other primates are expected to be benign in humans. In fact, among human variants with high quality benign/likely-benign or pathogenic/likely-pathogenic classification calls in ClinVar (2 stars or higher), that are also seen in other primates, 99% are benign/likely-benign [49]. For variants observed as homozygotes in other primate species, the expectation of benignity in humans is even stronger. On this basis, we examined the behavior in our assay of the 41 tested variants that have been observed as homozygotes in at least one non-human primate. This group behaved as expected for benign variants, occurring only at the upper part of our growth range, with the lowest exhibiting 84% growth (Fig 5). Interestingly, several primAD variants occurred at conserved residues (ConSurf<0), consistent with our data showing that many amino acid substitutions at these residues do not strongly impair protein function (S8 Fig). Including the 41 homozygous primAD variants in our analysis greatly improved our power to define the distribution of benign variants across our assay range relative to using only the two variants with ClinVar benign/likely-benign annotation. In particular, including the primAD data greatly reduced the size of the uncertain range of our assay (82–84% growth vs 82–91%), allowing us to reclassify 240/280 variants previously falling in the uncertain range as non-deleterious (Fig 5).

### Experimental models of homozygous and compound heterozygous diploid genotypes

**Fig 6:**
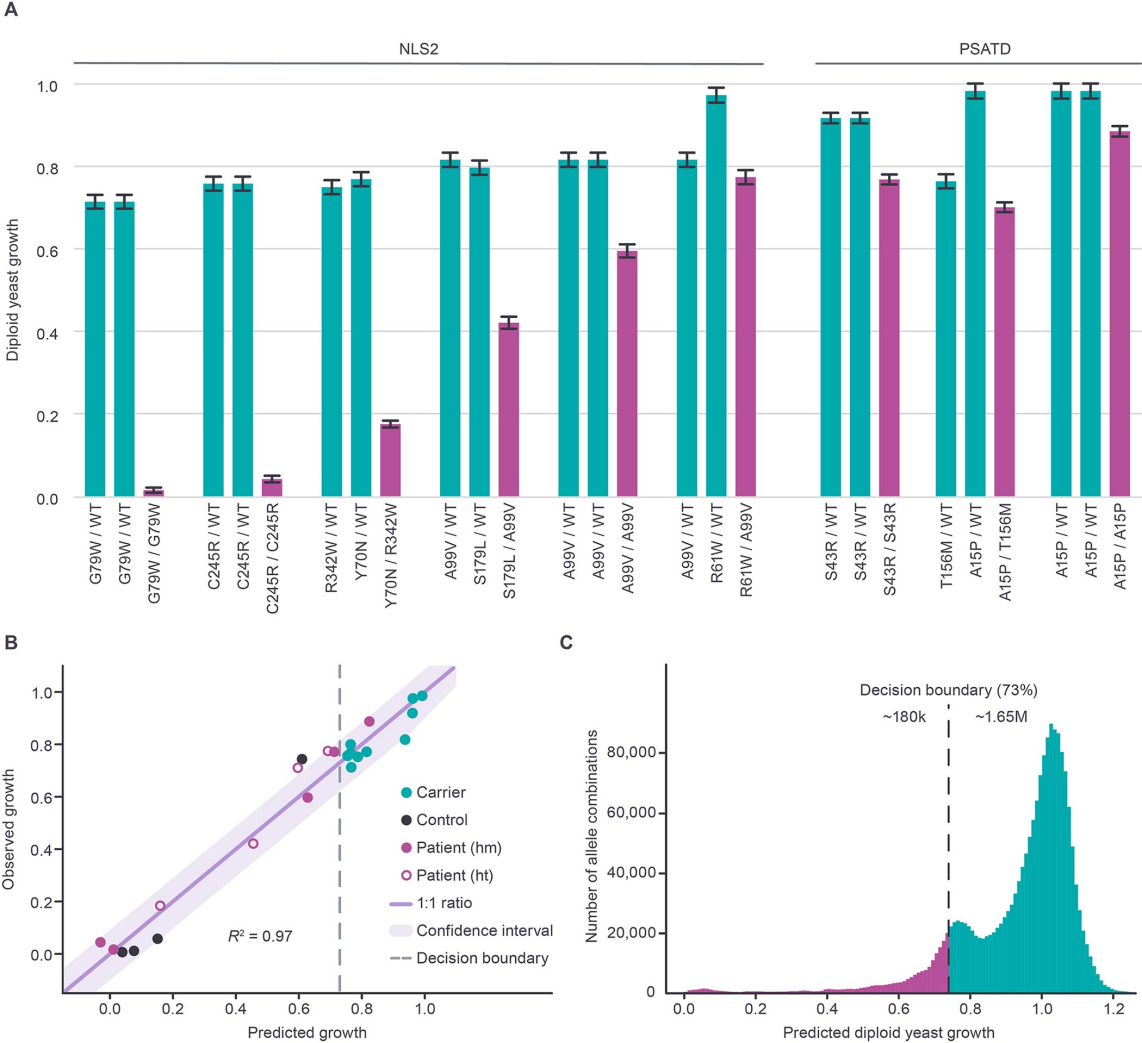
Modeling PSAT function in biallelic combinations. **(A)** Diploid yeast models of patient-parent trios. Bar charts depict mean normalized growth estimate (relative to homozygous wild type *yPSAT1/yPSAT1* and null/null, with error bars representing standard error. Carrier parents are shown in blue, and the offspring in purple. Recurrent carrier genotypes are repeated for ease of reference relative to the offspring. The clinical severity (NLS2 or PSATD) associated with patient genotypes is indicated above. **(B)** Observed versus fitted values shown for the pairwise additive linear regression model for predicting diploid growth (allele pairs) as a function of haploid growth (single alleles). Each circle represents a unique biallelic combination. Patient (homozygous or hm, heterozygous or ht), carrier, and control strains are labeled as indicated. A diagonal line of perfect correspondence (1:1 ratio) and the coefficient of determination (R^2^) are also included. The decision boundary for predicted diploid growth (73%) as a binary classifier for identifying patient genotypes modeled in our diploid assay is shown as a vertical dotted grey line. The confidence interval (95%) is shaded as purple. **(C)** Frequency of predicted diploid growth values (in 1% bins) for all ~1.8M combinations of alleles. The decision boundary of 73% growth is shown as a black dotted line.

Pathogenic variants in *PSAT1* cause disease that ranges from severe (Neu-Laxova syndrome 2, NLS2 [MIM: 616038]) to milder forms (PSAT deficiency, PSATD [MIM: 610992]). These diseases are collectively referred to as NLS2/PSATD (S1 Fig), or individually when clinical severity is discussed specifically. Because NLS2/PSATD is an autosomal recessive disease, clinical manifestation depends on the enzymatic function conferred by the combination of the two *PSAT1* alleles in an individual’s genome. The ability to generate stable diploid yeast cells by mating haploids allows us to easily generate diploid human *PSAT1* genotypes in yeast and quantitively assess the function of allele pairs. As in our previous study [26], we used our growth assay to functionally assess pairwise combinations of protein-coding variants across all reported unique patient (and carrier parent) genotypes available at the time of writing (S4 Table). Since our previous study [26], six additional reports [15,18,50–53] have added descriptions of 20 new NLS2/PSATD patients, eight new missense alleles and twelve unique genotypes to the disease literature (S4 Table). Each of the eight new disease alleles showed functional impairment in our assay, falling in the deleterious range defined above (S10 Fig). To experimentally measure the functional impact of the patient allele combinations in our yeast assay, we constructed diploid strains harboring *yPSAT1* allele combinations encoding the same pairs of PSAT amino acid sequences as the human genotypes. We then assayed the growth of these strains relative to homozygous wild type (*yPSAT1*) and homozygous null (*ser1*Δ0) strains on minimal medium lacking serine. Previously constructued *yPSAT1* diploid strains [26] encoding homozygous p.Ala99Val or p.Ser43Arg, and the compound heterzygote p.Ala99Val / p.Ser179Leu, were also included for comparision. As expected, diploid strains harboring patient genotypes (Fig 6A, purple bars) all showed significantly impaired growth, relative to the homozygous wildtype. Strains harboring carrier genotypes (Fig 6A, blue bars) displayed higher levels of growth, with only a small degree of overlap between the estimated patient and carrier growth distributions. In particular, each modelled patient genotype (S5 Table) displayed reduced growth relative to both of its specific carrier parents (Fig 6A). Consistent with our previous study [26], we also observed good agreement between the degree of *PSAT1* functional impairment in our diploid assay and disease severity (Fig 6A). Diploids corresponding to genotypes from NLS2 patients had normalized growth from 0% to 77%, while diploids corresponding to genotypes from PSATD patients had a higher normalized growth range of 70% to 88%.

Our assay results are also consistent with clinical stratification within the NLS2 and PSATD groups. For PSATD, the p.Ala15Pro / p.Ala15Pro homozygous genotype is the least impaired in our assay (88% growth) and is also the mildest form of PSATD reported to date. A recent report [15] described two unrelated individuals with this allele combination who were developmentally normal, exhibited ichthyosis, and presented with peripheral neuropathy at the ages of 16 and 17. For NLS2, the two genotypes with the lowest growth in our assay (p.Gly79Trp / p.Gly79Trp and p.Cys245Arg / p.Cys245Arg) correspond to individuals displaying severe NLS2 [18]. Three of the remaining NLS2 genotypes modelled here (p.Tyr70Asn / p.Arg342W, p.Ala99Val / p.Ser179Leu, p.Ala99Val / p.Arg61Trp) display higher growth in our assay and correspond to individuals described as presenting with moderate NLS [16,18,50]. Among these, p.Tyr70Asn / p.Arg342Trp was the most impaired in our assay (18% growth), and the affected individual had a postnatal survival of 8 weeks [50]. In contrast, the individual harboring p.Arg61Trp / p.Ala99Val (77% growth, least impaired) had the longest NLS2-associated postnatal survival described to date (4 months) [18]. Individuals homozygous for the p.Ala99Val genotype (60% growth) exhibit variable postnatal survival (ranging from 1 day to 9 weeks) [16–18]. Together, these results highlight the potential value of our model organism assay for variant interpretation in the context of diploid *PSAT1* genotypes.

### Predicting biallelic effects from individual allele measurements

Experimentally assessing the level of PSAT activity associated with all allele-pairs would require construction and assay of 1.83 million diploid yeast strains. As a more labor and cost-effective alternative, we evaluated whether a relationship exists between individual haploid estimates and their resulting diploid growth in combination. For homozygous diploids, a very strong linear relationship was seen between haploid and diploid growth estimates (S11 Fig; R^2^ = 0.98). To extend this analysis to heterozygous genotypes, we fit a model to our experimental diploid dataset of both patient and carrier genotypes (n = 23), to predict diploid growth as a linear combination of the allele with the higher and the allele with the lower haploid growth values (Fig 6B). The resulting model explained 97% of the variance in the growth of the diploids. This model assigned a greater weight to the higher growing allele than the lower growing allele (coefficients of 0.73 vs 0.28), consistent with incomplete dominance of the higher growing allele, and included a small positive intercept (0.05), reflecting a consistent small increase in variant growth in diploids relative to haploids. To compare how this model performed relative to alternatives with simpler assumptions, we also generated models that assumed either complete dominance of the higher-growing allele or an equal contribution of both alleles (S12 Fig). Our full model performed significantly better than either of the alternative models (Anova, Df = 1; p = <5.4x10^-6^ and p <2.3x10^-6^, respectively). Leave one out cross validation of the pairwise model displayed an excellent ability to predict diploid growth from haploid measurements (RMSE = 0.0698 and MAE = 0.0566).

We next compared the results of applying logistic regression to the predicted diploid growth values to produce a binary classifier capable of distinguishing the low growing genotypes of NLS2/PSATD patients (n = 9) from the high growing carrier parent genotypes in our trio models (n = 10). The threshold growth value at the decision boundary (p = 0.5) was 73% for predicted diploid growth and misclassified only one patient genotype (p.Ala15Pro / p.Ala15Pro) (Fig 6B). These results suggest that diploid growth values predicted from haploid measurements perform well in clinical classification of human *PSAT1* genotypes. On this basis, we computed the predicted diploid growth from all unique pairwise combinations (~1.8 million) of haploid estimates from 1,914 *yPSAT1* missense alleles, wild type (*yPSAT1*), and null (*ser1*Δ0) using the pairwise-additive model (Figs 6C and S13). We then classified whether the resulting values fell above or below the disease classification boundary of 73% growth. A large majority (89%) of genotypes fell above the decision boundary threshold, suggesting these genotypes would not lead to NLS2/PSATD (Figs 6C and S13). However, 177,760 *yPSAT1* biallelic combinations displayed predicted diploid growth below the classification threshold, consistent with these genotypes resulting in disease (Fig 6C).

## Discussion

Here, we present the results of a yeast-based assay for human PSAT activity applied at scale. The resulting dataset allowed us to comprehensively analyze the individual functional impact of 1,914 amino acid substitutions, representing 88% of all amino acid changes accessible by a missense SNV in the human *PSAT1* sequence. Compared to our previous study [26], which focused on 200 alleles, this larger, more densely sampled dataset allowed us to address new questions about PSAT biology. By assaying multiple amino acid substitutions at each residue of PSAT, we were able to explore protein structure-function relationships in detail. Consistent with the expectation that cofactor binding is required for catalysis, PLP binding residues were sensitive to the effect of most amino acid substitutions. In addition, we were able to use functional evidence to address limitations of the currently available crystallographic studies of human PSAT. The crystal structure of human PSAT was solved in the absence of bound substrate, however our data suggest that four of the five substrate-binding residues identified from the structures of orthologous proteins (solved with bound substrate) may also be important for substrate coordination in human PSAT. The solved crystal structure of human PSAT also lacks the N-terminal region of the protein, which is known to be important in some orthologs. This region contains a domain that AlphaFold predicts is proximal to the active site of the enzyme and our data suggest is important in the function of the human protein.

The densely sampled set of amino acid substitutions also allowed us to explore the relationship between evolutionary conservation and protein function across the full length of PSAT. Consistent with expectation, we observed that most amorphic substitutions occurred at conserved residues and that most substitutions at weakly conserved residues were non-deleterious in our assay. However, this analysis also demonstrated that many amino acid substitutions at conserved residues showed little or no growth impairment in our assay. Consistent with previously noted limitations of computational methods that utilize evolutionary conservation metrics [47], several frequently used predictors made false-positive predictions that PSAT variants would be deleterious. This result further emphasizes the importance of large-scale functional assays, such as the one described in this study, in accurately characterizing the effect of sequence variation on protein function.

While our assay is a powerful tool to study the effects of amino acid substitutions on PSAT function, the experimental design does have some limitations. As a generalized readout of protein function, our assay does not distinguish whether reductions in protein activity, caused by amino acid substitutions, are the result of changes in protein abundance, enzyme kinetics, or a combination of both. Similarly, because our yeast construct, *yPSAT1*, was designed to measure the effect of amino acid substitutions, our assay does not assess the impact of nucleotide variants that may affect transcription, splicing, or mRNA stability. As such, the results presented here will benefit from future studies that integrate orthogonal types of molecular and biochemical data in humans and model systems.

The 200 amino acid substitutions assayed in our previous study [26], represented all missense variants identified in the human population at the time. In contrast, the dataset presented here represents 88% of all amino acids substitutions that could arise via an SNV in *PSAT1*. As such, this new dataset has the potential to inform clinical interpretation for the great majority of all *PSAT1* missense variants that are likely to ever be discovered in the human population. Leveraging functional data for clinical variant interpretation requires an understanding of the relationship between protein function in the assay and clinical presentation. Our dataset shows clear stratification of existing clinical variants, with benign variants exhibiting little to no functional impairment, and pathogenic variants exhibiting substantial loss of activity. Similarly, variants observed as homozygotes in other primates show little functional impairment while variants from the disease literature display substantial reductions in activity. On this basis, we used the small number of variants with existing interpretation to provide evidence for the likely clinical impact of the large number of unclassified variants in similar ranges of the assay.

Methods have also been proposed to quantify the uncertainty associated with pathogenic versus benign classification calls [48]. However, as is the case for other rare and ultrarare genetic diseases, the small number of *PSAT1* variants available to inform classification boundaries limited the types of statistical approaches that could be used in our study. As additional clinical information becomes available through identification of new patients and additional research into the natural history of the disease, we expect that the ability to resolve the relationship between our functional dataset and the clinical impact of variants will improve. In fact, in the three years since our original study [26], additional patients were described in the disease literature. Not only was there good agreement between the results of our assay and disease severity in these individuals, but this additional clinical information also allowed us to extend the range of our assay that we could associate with clinical outcomes.

Monogenic autosomal recessive diseases, such as NLS2/PSATD, are a function of the pairwise allele combination present in each diploid human genome. In our previous study [26], we demonstrated that human *PSAT1* genotypes could be modeled in diploid yeast and that this growth assay could be used to experimentally assess the function of each allele pair. In the current study, we extended this analysis to include six recently published family trios (Fig 6A). Our results confirmed that patient genotypes showed more impaired PSAT activity than those of carrier parents and that NLS2 patient genotypes were more strongly impaired than PSATD genotypes. In addition, our results agreed with the degree of disease severity seen at a finer scale within the NLS2 and PSATD classes. Therefore, the quantitative experimental results of our diploid assay agree well with the clinical stratification of characterized human *PSAT1* genotypes. In particular, our results successfully distinguish disease-associated from non-disease allele combinations. The identified ranges of the assay correlating with healthy carriers and with different degrees of disease severity allow the likely clinical manifestation of uncharacterized genotypes to be predicted using our functional assay.

Information about the impact of allele combinations has the potential to significantly increase the diagnostic value of our functional dataset. However, experimentally measuring all possible combinations of our 1,914 *PSAT1* alleles (1.8 million potential human genotypes) is a substantial endeavor, even with a high-throughput yeast assay. We therefore developed a computational model that allows us to accurately predict the growth of allele pairs, in diploids, as a linear combination of the growth values of each allele, measured singly in haploids. As such, we can leverage the relatively small set of experimentally tested combinations used to fit the model, along with our single allele (haploid) dataset, to infer the growth of the full set of 1.8 million potential allele combinations. We also identified a classification boundary within the predicted growth range that accurately classifies which allele pairs are likely to be associated with disease.

Serine biosynthesis disorders, especially if diagnosed early, can be treated by serine and glycine supplementation [9–13]. Therefore, prior knowledge of whether an individual’s combination of alleles has the potential to cause disease can positively impact patient care. Large scale inferences of PSAT activity arising from allele combinations could also inform studies aimed at better capturing the full range of phenotypes associated with serine biosynthesis defects. For example, evidence has recently been presented that defects in enzymes of the serine biosynthesis pathway are associated with a rare neurovascular degenerative retinal disease (macular telangiectasia type 2) [54,55]. In combination with ongoing efforts like the All of US research program and the UK biobank [56] that integrate genome sequence and detailed biomedical traits, the ability to stratify individuals by the level of their serine biosynthesis activity could enable the discovery of associations between this pathway and additional diseases. Such associations would not only identify new causes of inherited disease, but also suggest potential treatments in the form of serine supplementation.

## Methods

### Strain library construction

All *Saccharomyces cerevisiae* strains used in this study (S1 and S2 Tables) were derived from the isogenic lab strains FY4 (MAT**a**) and FY5 (MATα) [57]. Unless otherwise noted, strains were grown in rich medium (YPD, 1% yeast extract, 2% peptone, and 2% glucose) or minimal medium (SD, without amino acids, 2% glucose) using standard media conditions and methods for yeast genetic manipulation [58].

The design and construction of the yeast codon-optimized version (*yPSAT1*) of human *PSAT1* isoform 1, the wild-type (*yPSAT1*) strain, and the deletion (*ser1*Δ*0)* strain were previously described in Sirr *et al*. [26]. The design, construction, and assay of the comprehensive library of SNV-accessible amino acid substitutions used in this study was performed as follows:

The variant library was designed to capture all amino acid substitutions across 369 codons (the full length of the protein excluding the start and stop codons) that could result from an SNV in the human *PSAT1* isoform 1 cDNA sequence (Consensus Coding Sequence database [59]: CCDS6660.1). The nine possible single nucleotide variants at each codon resulted in 4–7 unique amino acid substitutions. *yPSAT1* variants encoding the complete set of these unique amino acid substitutions (n = 2,182) were synthesized (Twist Biosciences) in *yPSAT1* as a variant library in which each well of a 96 well plate contained all amino acid substitutions (n = 4–7) at a given amino acid. Hereafter, we refer to this mixture of DNA fragments encoding variants at a single codon as a “single codon pool”.

Individual wells of these plates were amplified to approximately 500 ng of DNA by 15-cycles of high-fidelity PCR (Phusion High-Fidelity DNA Polymerase, Thermo Scientific) and transformed into a MAT**a** haploid deletion strain (*ser1*Δ*0*) using standard methods. Single colonies from the yeast transformations were isolated such that 6,335 individual transformants, each encoding a single amino acid substitution, were arrayed into 96-well plates containing rich medium. Additionally, as each colony was isolated, the information about which single codon pool it originated from was recorded. Thus, prior to sequencing the *yPSAT* construct in each strain, we know codon was targeted, but not the identity of the specific variant. Because individual transformants are isolated and maintained as separate stocks (one strain per well in a 96-well plate), each strain is an independently constructed biological isolate of the variant it contains. For downstream phenotype normalization, each library plate also contained replicates of the same control strains: 2 deletion (*ser1*Δ*0)* and 4 wild type (*yPSAT1*) strains.

### Variant library sequence confirmation

As described above, for each original *yPSAT1* transformant, the codon that is mutated (target codon) is known, but the identity of the specific variant is not. To determine this, we used a custom MinION (Oxford Nanopore Technologies) sequencing pipeline (S1 Text). Briefly, individual transformants were pooled in groups of 12, so that no target codon was represented more than once in a single pool. Each pool was then sequenced and, at each target codon, the most frequent potential variant codon was identified (candidate variant) as well as the second most frequent variant. Because we know which target codon corresponds to which transformant, this allows us to associate each candidate variant with a single transformant.

For any given DNA sequence, the frequency and pattern of MinIon sequencing errors varies greatly from base to base. These errors can occur at frequencies high enough to generate spurious matches to variant codons. However, the error patterns are also reproducible, allowing us to develop an error model for each variant, describing the frequency with which it is generated by sequencing errors. This frequency can be compared to the frequency observed for each candidate variant in the pooled sequencing, allowing true variants to be distinguished from sequencing noise [60].

Using this approach, candidate variants underwent quality control. Candidates were rejected if the observed frequency of the variant was less than 3.3x the estimated error frequency for that variant. In addition, candidates were also rejected if the second most frequent variant was both enriched (> = 10-fold) relative to its error frequency and was observed at >30% of the frequency of the candidate variant. Finally, candidates were rejected if they were supported by less than 15 reads, or if any missense or nonsense secondary mutations were present in the *yPSAT1* sequence. The end result of this process was that each transformant was assigned either a high confidence call for the variant present in that transformant, or an NA call that resulted in that transformant being removed from analysis. The script carrying out the steps for identifying the variant codon and any secondary mutations in each transformant, using Oxford Nanopore sequencing data, is provided as S1 Method.

The Sequence Read Archive accession number for MinION read sequences generated for this study is PRJEB60698.

### Generation of yeast diploids

Unless noted, all individual *yPSAT1* variants are present in haploid yeast strains with a single mating type (MAT**a**) (S1 Table). To generate diploid strains harboring combinations of *yPSAT1* alleles (missense variant, wild type, or null), variants were first introduced into a strain of the opposite mating type. The resulting *yPSAT1* MATα strains were then mated in a pairwise manner to the relevant *yPSAT1* MAT**a** strains using standard methods [61]. The resulting diploid strains, harboring combinations of *yPSAT1* alleles (S2 Table), were then arrayed in an alternating checkerboard pattern that minimizes the influence of nutrient competition from neighboring colonies during phenotype measurement.

### Growth assays

Before phenotyping, the set of variant strains generated in this study were extended to include the haploid strains carrying missense variants constructed and sequence confirmed by Sirr *et al* [26]. These additional strains, including at least 2 isolates of each variant, were re-arrayed to match the phenotyping layout used in this study.

A Biomek i7 robot outfitted with a V&P 96-pin head was utilized to pin strain plate libraries between different culture media. Strains were initially grown to saturation in rich liquid medium and then pinned onto solid medium utilizing glycerol as the central carbon source (YPG,1% yeast extract, 2% peptone, 2% glycerol) to remove any respiratory-deficient yeast cells. Strains were then pinned back into rich liquid medium and grown to saturation. Each plate was then pinned in replicate (n = 3–6) onto solid minimal medium, which lacks serine, and grown for 3 days at 30°C. A mounted Canon PowerShot SX10 IS compact digital camera was used to take images (ISO200, f4.5, 1/40s exposure) every 24 hours for three days under consistent lighting, camera to subject distance, and zoom. Each plate was labeled with a custom barcode that was included in the frame of view. Images were acquired as jpg files.

### Image-based growth quantification

Each plate image was processed using PyPl8 (https://github.com/lacyk3/PyPl8) to extract features from each strain patch in each barcoded agar plate as follows. First, the barcode within each image was detected and decoded to rename each file using the corresponding plate name, replicate number, condition, and timepoint. Next, each image was cropped into 96 square tiles, segmented, and each replica pinned patch was identified using Otsu’s method or circle detection. Finally, the sum of the gray scale pixel intensities within each strain patch (pixelsum), was extracted and used as the metric for growth estimation.

### Growth data fitting and normalization

For haploid strains, raw phenotypic values were normalized, quality control filters were applied to each isolate, and a final relative growth estimate for each variant was determined, as described in Lo *et al*. [60]. Briefly, normalization steps were carried out to account for the effects of plate-to-plate variation, relative growth of neighboring patches, and plate edge effects. Pin effect normalization did not reduce noise and was omitted. Isolates with (nonsynonymous) secondary mutations were removed from the dataset as were all isolates of variants that showed a high degree of variation in isolate-to-isolate growth values. This left a final filtered dataset of 5,164 independent isolates. Finally, a linear model was used to estimate the relative growth of each genotype, on a scale with growth of null controls set to 0 and growth of wild type set to 1. The script carrying out the growth normalization steps is provided as S2 Method.

A similar approach was applied to phenotypic values extracted from the diploid growth assay, although neighbor effects were assumed negligible because of the checkerboard pinning arrangement. For normalization, a linear model was used to simultaneously estimate the effects of genotype, plate edge positioning and plate-to-plate variation on growth. Genotype effects were rescaled to set homozygous null to 0 and homozygous wild type to 1.

### Predicting diploid growth

We developed a linear model to predict the growth of diploid strains based on which pair of *yPSAT1* variants is present, using individual growth estimates of each allele in haploids. In this model, for each diploid, the (haploid) growth estimates of the lower and higher growth alleles were labelled as minimum and maximum (*j,k*), respectively. In cases of homozygous combinations, the minimum and maximum were equal. Strains carrying a single copy of the wild type allele (*yPSAT1*) or null (*ser1*Δ0) had their respective haploid estimates set equal to 1 and 0. To predict diploid growth (d) from the more impaired (*x_j_*) and less impaired (*x_k_*) alleles, we performed an ordinary least squares regression to fit an additive pairwise model (d = a + b*x_j_* + c*x_k_*), with resulting coefficients being a = 0.05, b = 0.28, and c = 0.73. We also compared this model to two simpler regression models. In the first model, diploid growth was predicted from the mean of the haploid estimates (b = c). In the second, diploid growth was predicted from the higher growing haploid allele only, i.e. complete dominance (b = 0). Leave one out cross validation was used to assess model performance in the main model.

Next, we evaluated the performance of experimental versus predicted growth as a binary classifier for identifying genotypes matching those of individuals diagnosed with *PSAT1*-related serine biosynthesis defects vs carrier parents. We fit a logistic regression model to predicted diploid growth values (Log(p/1-p) = g + h*y_p_*) calculated from the additive pairwise model. The resulting coefficients for the regression model were g = -20.0 and h = 27.2. The logistic regression model indicated a threshold (p = 0.5) decision boundary at 73% predicted diploid growth.

### Protein structure and conservation analysis

Structural features were derived from the crystal structure of human phosphoserine aminotransferase isoform 1 complexed with pyridoxal phosphate cofactor (PDB: 3e77; subunit Leu17-Leu370, homodimer biological assembly). Secondary structure features were extracted from this crystal structure using DSSP software [62,63]. AlphaFold (version 2022-11-01) [43,44] was used to predict the structure of the missing 16 N-terminal residues. The PLP cofactor was transplanted into the AlphaFold predicted structure using AlphaFill [64]. Molecular visualizations were created using the PyMol Molecular Graphics System Version 2.3.2 (Schrödinger). Tools from the publicly available PyMol script and plugin repository were used to determine interfacial residues and charge distribution. Dimer interface residues were defined as residues at which the solvent-exposed surface area in the monomeric model is greater than the solvent-exposed surface area in the dimer model (cutoff value = 0.5 Å^2^). Macromolecular electrostatics were estimated for each residue of PDB 3e77 using the Adaptive Poisson-Boltzmann Solver PyMol plugin. Evolutionary conservation scores and ‘grades’ for each position of the full-length amino acid sequence of human PSAT (UniProt [65]: Q9Y617-1) were computed using ConSurf [46].All parameters for the ConSurf calculation were the same as the methodology outlined in creating the ConSurf-DB repository [66], with the best evolutionary model determined to be WAG [67]. The evolutionary conservation scores and ‘grades’ represent the calculated positional conservation based on an amino acid alignment of 300 diverse homologs of PSAT. The determined structure and conservation features for each amino acid position are provided in S6 Table.

## Supporting information

S1 Fig Key developments in the study of serine biosynthesis defects. (DOCX)

S2 Fig Overall subunit organization and homodimer assembly of human PSAT. (DOCX)

S3 Fig Distribution of variant growth scores at conserved substrate binding residues. (DOCX)

S4 Fig Full-length PSAT subunit structure as predicted by AlphaFold. (DOCX)

S5 Fig Median yeast growth versus evolutionary conservation per amino acid position in PSAT. (DOCX)

S6 Fig Variant effect prediction of PSAT missense variants. (DOCX)

S7 Fig Comparison of variants with ClinVar annotation to the distribution of functional scores, structure, and conservation for PSAT. (DOCX)

S8 Fig Comparison of PSAT functional and conservation scores relative to variants in ClinVar and primAD. (DOCX)

S9 Fig Allele frequency and yeast growth scores for PSAT1 missense variants found in gnomAD. (DOCX)

S10 Fig Haploid growth estimates of amino acid substitutions for missense alleles recently reported in the disease literature. (DOCX)

S11 Fig Haploid versus diploid yeast growth scores for alleles that are found in homozygous patient genotypes. (DOCX)

S12 Fig Comparing models for predicting diploid yeast growth representing biallelic combinations. (DOCX)

S13 Fig Heatmap of the predicted diploid growth value for all possible pairwise combinations of haploid allele measurements. (DOCX)

S1 Text Supplemental Methods. (DOCX)

S1 Table S. cerevisiae strains used in the haploid assay. (XLSX)

S2 Table S. cerevisiae strains used in the diploid assay. (XLSX)

S3 Table Normalized yPSAT1 haploid growth scores and standard error estimates with annotations in ClinVar, primAD, and the disease literature. (XLSX)

S4 Table Disease literature review of patients diagnosed with PSAT1-related serine biosynthesis defects. (XLSX)

S5 Table Normalized yPSAT1 diploid growth scores and standard error estimates. (XLSX)

S6 Table Human PSAT structure, conservation, and median haploid yeast growth data per amino acid position. (XLSX)

S7 Table Computational predictions for SNV-acessible PSAT missense variants. (XLSX)

S8 Table Multiplexed sequencing for MinIon run ONP025. (XLSX)

S9 Table Multiplexed sequencing for MinIon run ONP026. (XLSX)

S10 Table Multiplexed sequencing for MinIon run ONP027. (XLSX)

S11 Table Multiplexed sequencing for MinIon run ONP029. (XLSX)

S12 Table Multiplexed sequencing for MinIon run ONP028 and ONP030. (XLSX)

S13 Table Multiplexed sequencing for MinIon run ONP031. (XLSX)

S1 Method R script identifying the variant present in each yPSAT1 transformant, and any secondary mutations, from pooled Oxford Nanopore MinIon sequencing. (TXT)

S2 Method R script carrying out normalization steps for strain growth measurements. Normalization accounts for the effects of plate-to-plate variation, relative growth of neighboring patches, and plate edge effects.
(TXT)
