# Supplementary material for: Constructing and interpreting a large-scale variant effect map for an ultrarare disease gene: Comprehensive prediction of the functional impact of PSAT1 genotypes
Source: PLoS Genet. 2023 Oct 9;19(10):e1010972. doi: 10.1371/journal.pgen.1010972 (PMC10561871; doi:10.1371/journal.pgen.1010972)
Supplement: S1 Fig — (DOCX) [file pgen.1010972.s001.docx]

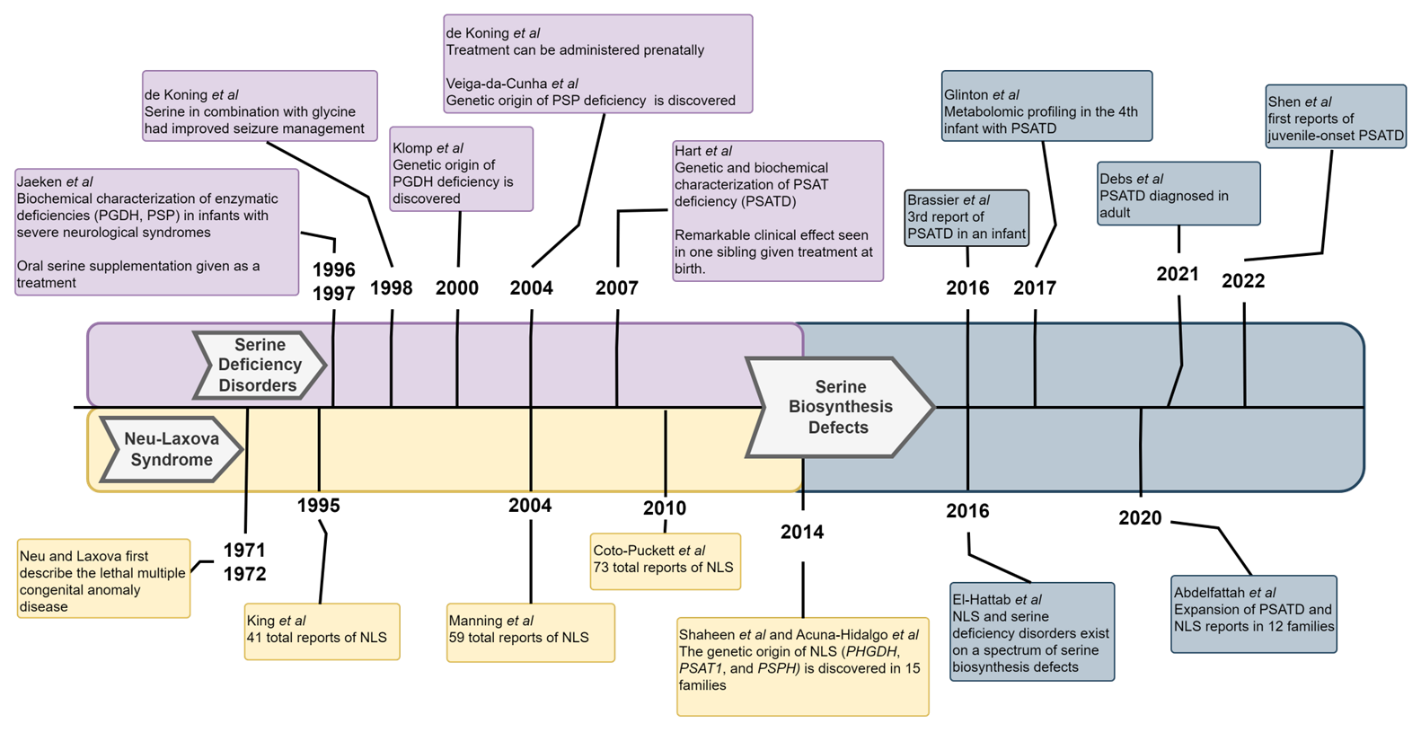


**S1 Fig. Key developments in the study of serine biosynthesis defects**. A timeline of milestones in the research and treatment of serine biosynthesis defects over the last three decades as described in the disease literature [1–20].

**Supplemental References**

1. Neu RL, Kajii T, Gardner LI, Nagyfy SF. A lethal syndrome of microcephaly with multiple congenital anomalies in three siblings. Pediatrics. 1971;47: 610–2.

2. Laxova R, Ohara PT, Timothy JA. A further example of a lethal autosomal recessive condition in sibs. J Ment Defic Res. 1972;16: 139–43. doi:10.1111/j.1365-2788.1972.tb01585.x

3. Jaeken J, Detheux M, Fryns JP, Collet JF, Alliet P, Van Schaftingen E. Phosphoserine phosphatase deficiency in a patient with Williams syndrome. J Med Genet. 1997;34: 594–596. doi:10.1136/jmg.34.7.594

4. Jaeken J, Detheux M, Van Maldergem L, Foulon M, Carchon H, Van Schaftingen E, et al. 3-Phosphoglycerate dehydrogenase deficiency: an inborn error of serine biosynthesis. Archives ofDisease in Childhood. 1996.

5. King JAC, Gardner V, Chen H, Blackburn W. Neu-laxova syndrome: Pathological evaluation of a fetus and review of the literature. Fetal Pediatr Pathol. 1995;15: 57–79. doi:10.3109/15513819509026940

6. De Koning TJ, Duran M, Van Maldergem L, Pineda M, Dorland L, Gooskens R, et al. Congenital microcephaly and seizures due to 3-phosphoglycerate dehydrogenase deficiency: Outcome of treatment with amino acids. J Inherit Metab Dis. 2002.

7. Klomp LW, de Koning TJ, Malingré HE, van Beurden EA, Brink M, Opdam FL, et al. Molecular characterization of 3-phosphoglycerate dehydrogenase deficiency--a neurometabolic disorder associated with reduced L-serine biosynthesis. Am J Hum Genet. 2000;67: 1389–99. doi:10.1086/316886

8. de Koning TJ, Klomp LWJ, Oppen ACCV, Beemer PFA, Dorland L, Berg IETV Den, et al. Prenatal and early postnatal treatment in 3-phosphoglycerate-dehydrogenase deficiency. Lancet. 2004;364: 2221–2222. doi:10.1016/S0140-6736(04)17596-X

9. Veiga-da-Cunha M, Collet J-F, Prieur B, Jaeken J, Peeraer Y, Rabbijns A, et al. Mutations responsible for 3-phosphoserine phosphatase deficiency. Eur J Hum Genet. 2004;12: 163–6. doi:10.1038/sj.ejhg.5201083

10. Manning MA, Cunniff CM, Colby CE, El-Sayed YY, Hoyme HE. Neu-Laxova Syndrome: Detailed Prenatal Diagnostic and Post-Mortem Findings and Literature Review. Am J Med Genet. 2004;125 A: 240–249. doi:10.1002/ajmg.a.20467

11. Hart CE, Race V, Achouri Y, Wiame E, Sharrard M, Olpin SE, et al. Phosphoserine aminotransferase deficiency: A novel disorder of the serine biosynthesis pathway. Am J Hum Genet. 2007;80: 931–937. doi:10.1086/517888

12. Coto-Puckett WL, Gilbert-Barness E, Steelman CK, Stuart T, Robinson HB, Shehata BM. A spectrum of phenotypical expression of Neu-laxova syndrome: Three case reports and a review of the literature. Fetal Pediatr Pathol. 2010;29: 108–119. doi:10.3109/15513811003620914

13. Shaheen R, Rahbeeni Z, Alhashem A, Faqeih E, Zhao Q, Xiong Y, et al. Neu-laxova syndrome, an inborn error of serine metabolism, is caused by mutations in PHGDH. Am J Hum Genet. 2014;94: 898–904. doi:10.1016/j.ajhg.2014.04.015

14. Acuna-Hidalgo R, Schanze D, Kariminejad A, Nordgren A, Kariminejad MH, Conner P, et al. Neu-laxova syndrome is a heterogeneous metabolic disorder caused by defects in enzymes of the l-serine biosynthesis pathway. Am J Hum Genet. 2014;95: 285–293. doi:10.1016/j.ajhg.2014.07.012

15. Brassier A, Valayannopoulos V, Bahi-Buisson N, Wiame E, Hubert L, Boddaert N, et al. Two new cases of serine deficiency disorders treated with l-serine. European Journal of Paediatric Neurology. 2016;20: 53–60. doi:10.1016/j.ejpn.2015.10.007

16. El-Hattab AW. Serine biosynthesis and transport defects. Molecular Genetics and Metabolism. Academic Press Inc.; 2016. pp. 153–159. doi:10.1016/j.ymgme.2016.04.010

17. Glinton KE, Benke PJ, Lines MA, Geraghty MT, Chakraborty P, Al-Dirbashi OY, et al. Disturbed phospholipid metabolism in serine biosynthesis defects revealed by metabolomic profiling. Mol Genet Metab. 2018;123: 309–316. doi:10.1016/j.ymgme.2017.12.009

18. Abdelfattah F, Kariminejad A, Kahlert AK, Morrison PJ, Gumus E, Mathews KD, et al. Expanding the genotypic and phenotypic spectrum of severe serine biosynthesis disorders. Hum Mutat. 2020;41: 1615–1628. doi:10.1002/humu.24067

19. Debs S, Ferreira CR, Groden C, Kim HJ, King KA, King MC, et al. Adult diagnosis of congenital serine biosynthesis defect: A treatable cause of progressive neuropathy. Am J Med Genet A. 2021;185: 2102–2107. doi:10.1002/ajmg.a.62245

20. Shen Y, Peng Y, Huang P, Zheng Y, Li S, Jiang K, et al. Juvenile-onset PSAT1-related neuropathy: A milder phenotype of serine deficiency disorder. Front Genet. 2022;13. doi:10.3389/fgene.2022.949038
