# Supplementary material for: Constructing and interpreting a large-scale variant effect map for an ultrarare disease gene: Comprehensive prediction of the functional impact of PSAT1 genotypes
Source: PLoS Genet. 2023 Oct 9;19(10):e1010972. doi: 10.1371/journal.pgen.1010972 (PMC10561871; doi:10.1371/journal.pgen.1010972)
Supplement: S2 Fig — (DOCX) [file pgen.1010972.s002.docx]

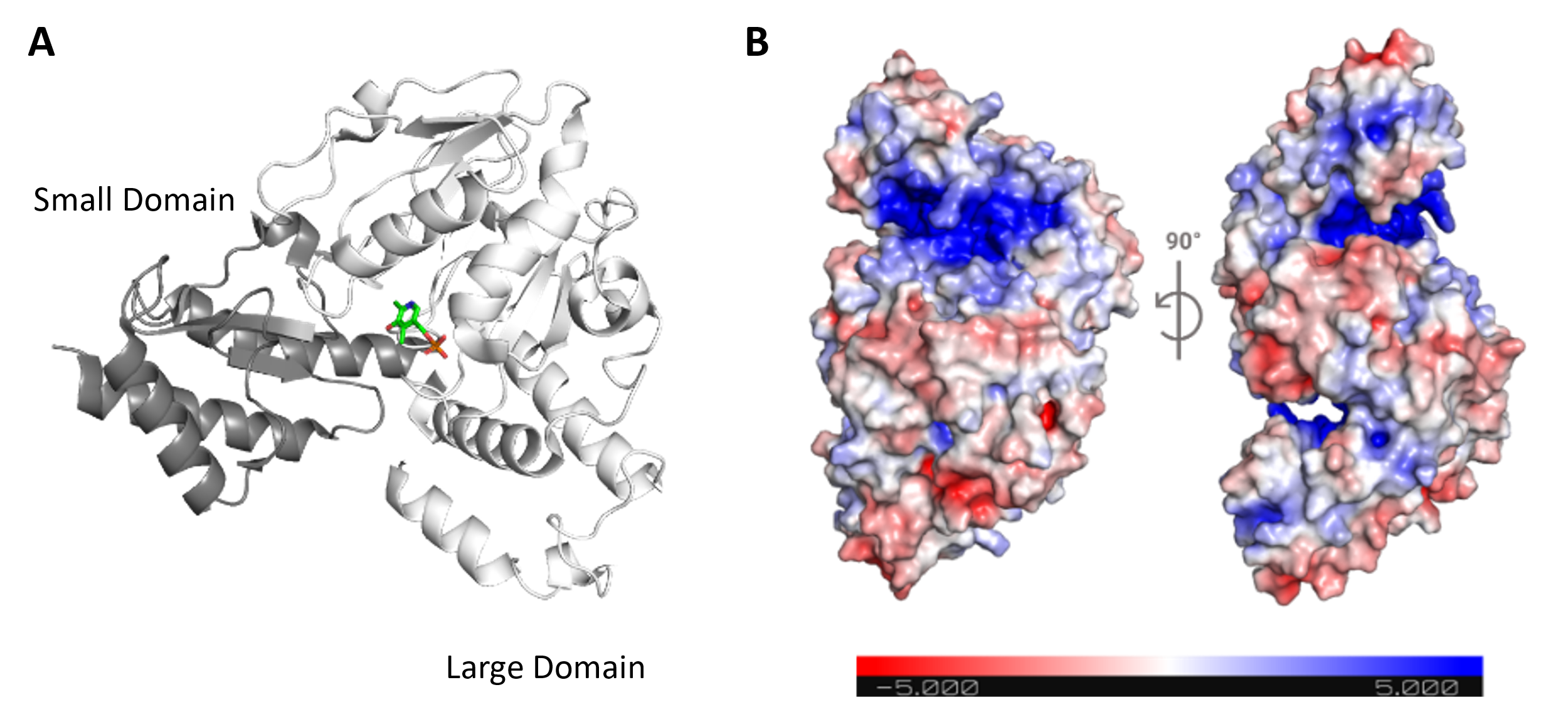


**S2 Fig. Overall subunit organization and homodimer assembly of human PSAT.** (A) Ribbon diagram representation of the secondary structure of a subunit of PSAT (PDB 3e77; Leu17-Leu370) complexed with PLP in stick representation colored by element (carbon: green, nitrogen: blue, oxygen: red, phosphate: orange). The small C-terminal domain is colored as dark grey and large N-terminal domain as white and labeled in (A). (B) Local charge distribution on the surface of the PSAT homodimer assembly, on a scale of negative charge (-5 kT; red) to positive charge (+5 kT; blue) as calculated by the Adaptive Poisson–Boltzmann Solver program.
