# Supplementary material for: Constructing and interpreting a large-scale variant effect map for an ultrarare disease gene: Comprehensive prediction of the functional impact of PSAT1 genotypes
Source: PLoS Genet. 2023 Oct 9;19(10):e1010972. doi: 10.1371/journal.pgen.1010972 (PMC10561871; doi:10.1371/journal.pgen.1010972)
Supplement: S3 Fig — (DOCX) [file pgen.1010972.s003.docx]

**
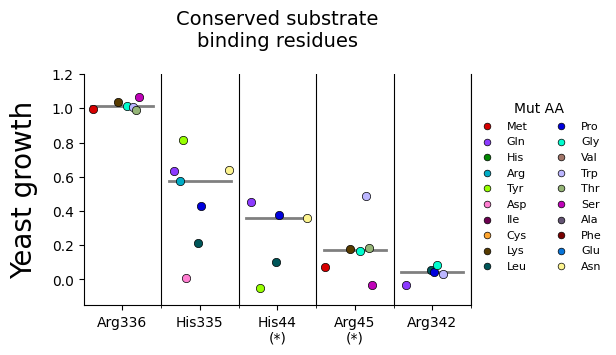
**

**S3 Fig**. **Distribution of variant growth scores at conserved substrate binding residues.** Strip plot of the yeast growth for each tested amino acid substitution at putative substrate binding residues previously identified in *Escherichia coli* [1], *Bacillus alcalophilus* [2], and *Arabidopsis thaliana* [3] phosphoserine aminotransferases that had crystal structures in phosphoserine or α-methyl-L-glutamate (analog) bound states. Each circle represents the normalized haploid yeast growth score (scaled relative to wild type *yPSAT1* (normalized growth=1) and null (normalized growth=0)), colored according to which amino acid substitution (legend; Mut AA) was tested. Grey horizontal bars represent the median growth estimate at that amino acid position. Asterisks indicate residues that are present on the opposite subunit in the functional homodimer assembly (PDB: 3e77).

**Supplemental References**

1. Hester G, Stark W, Moser M, Kallen J, Marković-Housley Z, Jansonius JN. Crystal structure of phosphoserine aminotransferase from Escherichia coli at 2.3 A resolution: comparison of the unligated enzyme and a complex with alpha-methyl-l-glutamate. J Mol Biol. 1999;286: 829–50. doi:10.1006/jmbi.1998.2506

2. Battula P, Dubnovitsky AP, Papageorgiou AC. Structural basis of l-phosphoserine binding to Bacillus alcalophilus phosphoserine aminotransferase. Acta Crystallogr D Biol Crystallogr. 2013;69: 804–811. doi:10.1107/S0907444913002096

3. Sekula B, Ruszkowski M, Dauter Z. Structural analysis of phosphoserine aminotransferase (isoform 1) from arabidopsis Thaliana– the enzyme involved in the phosphorylated pathway of serine biosynthesis. Front Plant Sci. 2018;9. doi:10.3389/fpls.2018.00876
