# Supplementary material for: Constructing and interpreting a large-scale variant effect map for an ultrarare disease gene: Comprehensive prediction of the functional impact of PSAT1 genotypes
Source: PLoS Genet. 2023 Oct 9;19(10):e1010972. doi: 10.1371/journal.pgen.1010972 (PMC10561871; doi:10.1371/journal.pgen.1010972)
Supplement: S4 Fig — (DOCX) [file pgen.1010972.s004.docx]

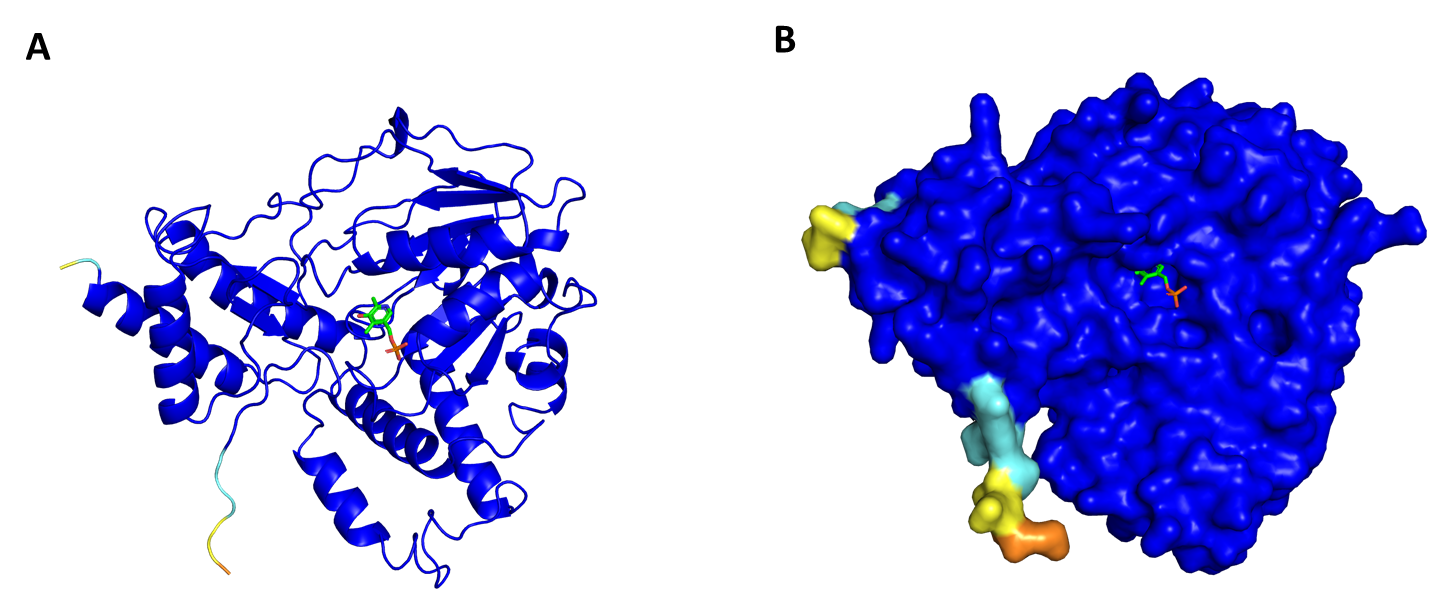


**S4 Fig. Full-length** **PSAT subunit structure as predicted by AlphaFold.** (A) Ribbon and (B) Surface representation of the full-length subunit structure for human PSAT as predicted by AlphaFold [1,2]. Residues are colored by the standard pLDDT confidence measure, with very confident (pLDDT >90) scores shown as dark blue, confident scores (90 > pLDDT > 70) as light blue, low scores (70 > pLDDT > 50) as yellow, and very low (pLDDT <50) scores as orange. The PLP cofactor is represented as a stick colored by element (carbon: green, nitrogen: blue, oxygen: red, phosphate orange), and was transplanted into the predicted structure using AlphaFill [3], to indicate the location of one of the active sites.

**Supplemental References**

1. Varadi M, Anyango S, Deshpande M, Nair S, Natassia C, Yordanova G, et al. AlphaFold Protein Structure Database: massively expanding the structural coverage of protein-sequence space with high-accuracy models. Nucleic Acids Res. 2022;50: D439–D444. doi:10.1093/nar/gkab1061

2. Jumper J, Evans R, Pritzel A, Green T, Figurnov M, Ronneberger O, et al. Highly accurate protein structure prediction with AlphaFold. Nature. 2021;596: 583–589. doi:10.1038/s41586-021-03819-2

3. Hekkelman ML, de Vries I, Joosten RP, Perrakis A. AlphaFill: enriching AlphaFold models with ligands and cofactors. Nat Methods. 2022. doi:10.1038/s41592-022-01685-y
