# Supplementary material for: Constructing and interpreting a large-scale variant effect map for an ultrarare disease gene: Comprehensive prediction of the functional impact of PSAT1 genotypes
Source: PLoS Genet. 2023 Oct 9;19(10):e1010972. doi: 10.1371/journal.pgen.1010972 (PMC10561871; doi:10.1371/journal.pgen.1010972)
Supplement: S5 Fig — (DOCX) [file pgen.1010972.s005.docx]

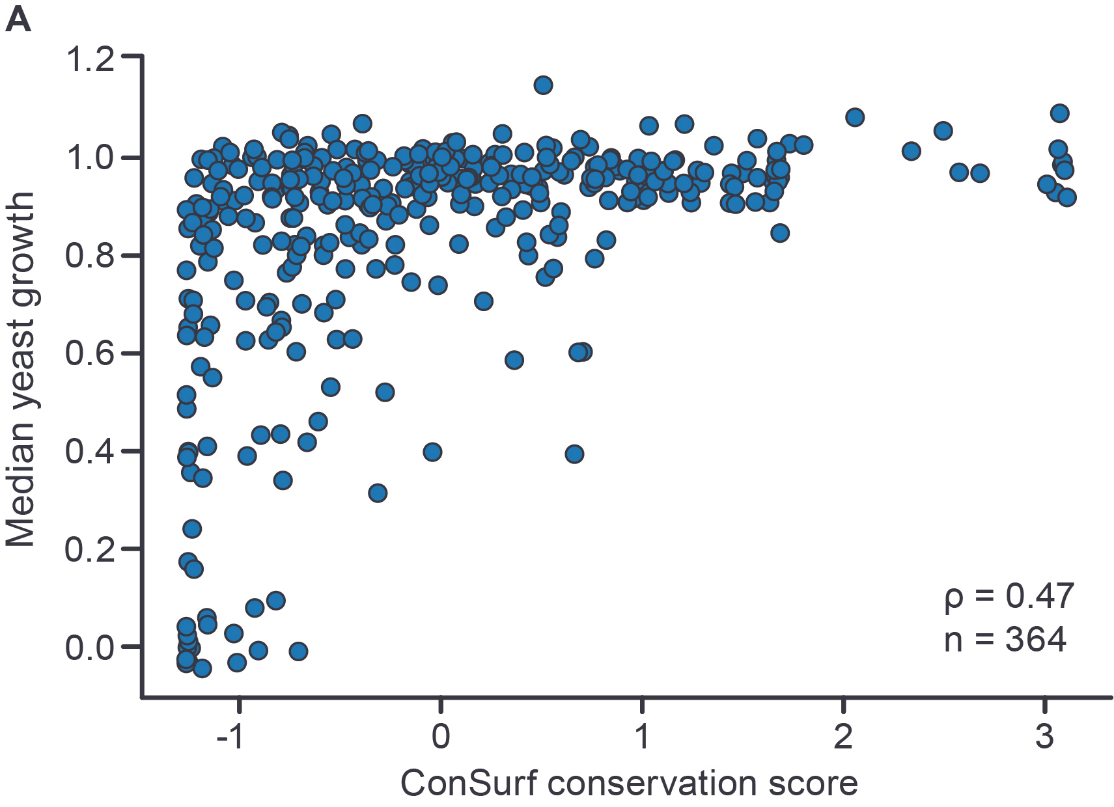


**S5 Fig. Median yeast growth versus evolutionary conservation per amino acid position in PSAT.** Scatterplot of the median yeast (haploid) growth score and ConSurf [1] conservation score for each corresponding amino acid position in human PSAT. More negative ConSurf scores indicate a higher degree of conservation. The corresponding Spearman rank correlation (ρ, pval<7.2 x 10^-22^), and number of residues with a ConSurf and median growth score (n) are labeled on the plot.

**Supplemental References**

1. Ashkenazy H, Abadi S, Martz E, Chay O, Mayrose I, Pupko T, et al. ConSurf 2016: an improved methodology to estimate and visualize evolutionary conservation in macromolecules. Nucleic Acids Res. 2016;44: W344-50. doi:10.1093/nar/gkw408
