# Supplementary material for: Constructing and interpreting a large-scale variant effect map for an ultrarare disease gene: Comprehensive prediction of the functional impact of PSAT1 genotypes
Source: PLoS Genet. 2023 Oct 9;19(10):e1010972. doi: 10.1371/journal.pgen.1010972 (PMC10561871; doi:10.1371/journal.pgen.1010972)
Supplement: S6 Fig — (DOCX) [file pgen.1010972.s006.docx]

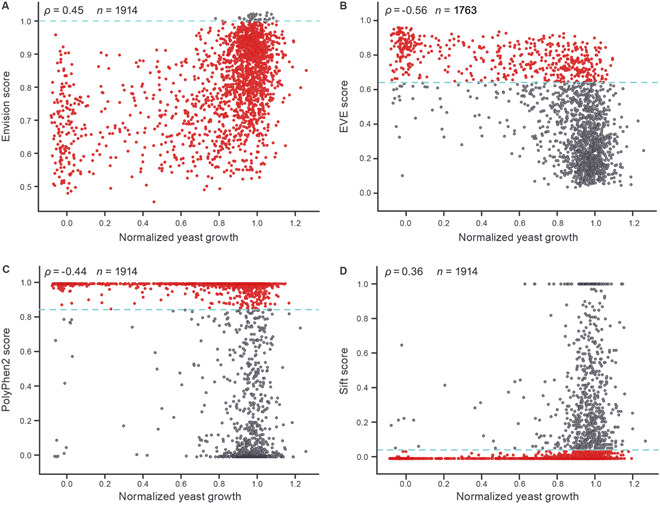


**S6 Fig. Variant effect prediction of PSAT missense variants.** Scatterplots of PSAT yeast (haploid) growth scores versus computational predictions. For all plots, correlation (Spearman rank, ρ) and the number of missense variants with predictor and yeast growth scores (n) are labeled. In (A), Envision [1] score versus normalized yeast growth. An Envision score <1 is considered damaging relative to wild type. In (B), EVE [2] score versus normalized yeast growth. An EVE score of <0.64 is considered damaging (EVE Class (75% Set). In (C), PolyPhen2 [3] score versus normalized yeast growth. A PolyPhen2 score >0.85 is considered damaging. In (D), SIFT [4] scores versus normalized yeast growth. A SIFT score <0.05 is considered damaging. Each predictor cutoff is shown as horizontal blue dotted lines. Variants that are considered damaging by the predictor cutoffs are colored red. All scores are provided in S7 Table.

**Supplemental References**

1. Gray VE, Hause RJ, Luebeck J, Shendure J, Fowler DM. Quantitative Missense Variant Effect Prediction Using Large-Scale Mutagenesis Data. Cell Syst. 2018;6: 116-124.e3. doi:10.1016/j.cels.2017.11.003

2. Frazer J, Notin P, Dias M, Gomez A, Min JK, Brock K, et al. Disease variant prediction with deep generative models of evolutionary data. Nature. 2021;599: 91–95. doi:10.1038/s41586-021-04043-8

3. Adzhubei IA, Schmidt S, Peshkin L, Ramensky VE, Gerasimova A, Bork P, et al. A method and server for predicting damaging missense mutations. Nat Methods. 2010;7: 248–9. doi:10.1038/nmeth0410-248

4. Sim N-L, Kumar P, Hu J, Henikoff S, Schneider G, Ng PC. SIFT web server: predicting effects of amino acid substitutions on proteins. Nucleic Acids Res. 2012;40: W452-7. doi:10.1093/nar/gks539
