# Supplementary material for: Constructing and interpreting a large-scale variant effect map for an ultrarare disease gene: Comprehensive prediction of the functional impact of PSAT1 genotypes
Source: PLoS Genet. 2023 Oct 9;19(10):e1010972. doi: 10.1371/journal.pgen.1010972 (PMC10561871; doi:10.1371/journal.pgen.1010972)
Supplement: S7 Fig — (DOCX) [file pgen.1010972.s007.docx]

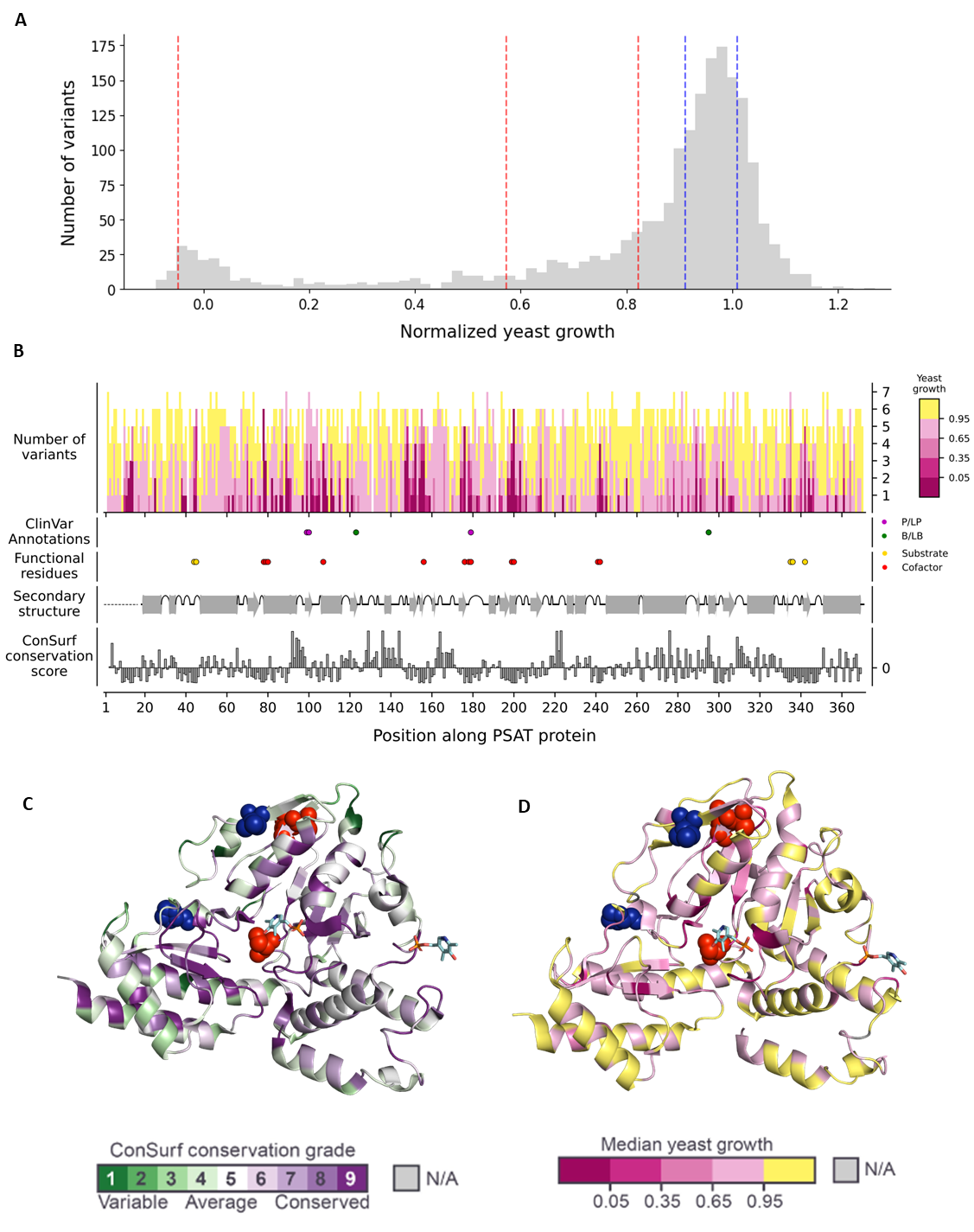


**S7 Fig**. **Comparison of variants with ClinVar annotation to the distribution of functional scores, structure, and conservation for PSAT.** (A) The distribution of growth scores (normalized relative to wild type *yPSAT1* and null) for 1,914 amino acid substitutions in PSAT (in 2% intervals) with growth values for ClinVar pathogenic/likely pathogenic and likely benign variants depicted by red and blue dashed vertical lines, respectively. (B) Variant effect map across the length of human PSAT with an additional layer showing the amino acid positions that have ClinVar pathogenic/likely pathogenic and likely benign variants that result in missense substitutions. Ribbon structures of human PSAT (PDB: 3e77) colored by their (C) ConSurf conservation grade and (D) median yeast growth score. The amino acid positions of ClinVar pathogenic/likely pathogenic and likely benign variants that result in missense substitutions are shown as red and blue spheres in (C-D), respectively. The PLP cofactor in (C-D) is shown in stick representation colored by element (carbon: cyan, nitrogen: blue, oxygen: red, phosphate orange).
