# Supplementary material for: Constructing and interpreting a large-scale variant effect map for an ultrarare disease gene: Comprehensive prediction of the functional impact of PSAT1 genotypes
Source: PLoS Genet. 2023 Oct 9;19(10):e1010972. doi: 10.1371/journal.pgen.1010972 (PMC10561871; doi:10.1371/journal.pgen.1010972)
Supplement: S8 Fig — (DOCX) [file pgen.1010972.s008.docx]

**
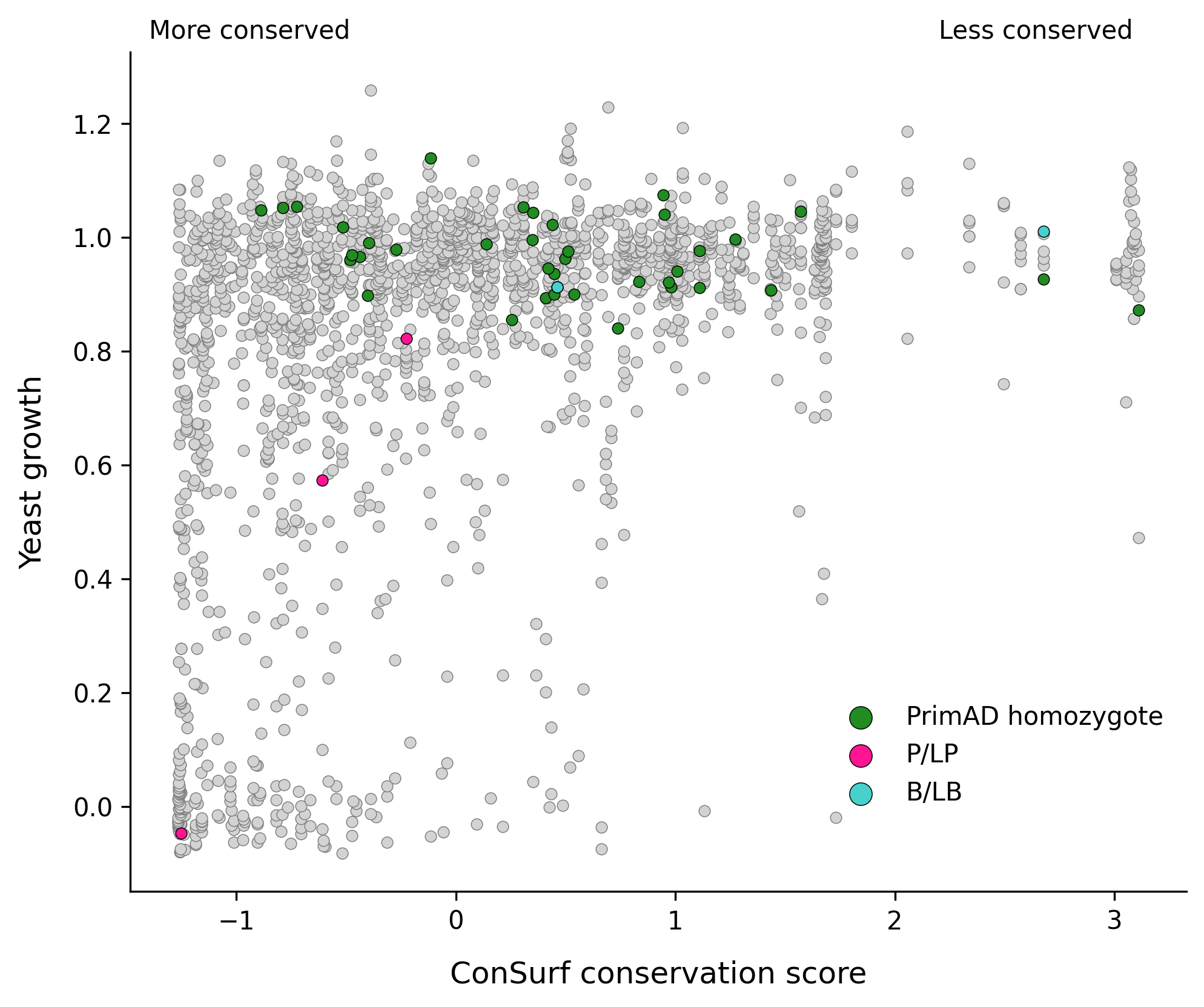
**

**S8 Fig**. **Comparison of PSAT functional and conservation scores relative to variants in ClinVar and primAD.** Scatterplot of all tested variants (n=1,902) ordered by their yeast growth score and ConSurf Conservation score. Variants annotated as pathogenic/likely pathogenic or likely benign in ClinVar are shown as pink and blue filled circles, respectively. Variants that are observed in primate homozygotes in primAD [1] are depicted as green filled circles. Grey filled circles indicate tested variants that do not fall into either of the three previous categories.

**Supplemental Reference**

1. Gao H, Hamp T, Ede J, Schraiber JG, McRae J, Singer-Berk M, et al. The landscape of tolerated genetic variation in humans and primates. Science. 2023;380: eabn8153. doi:10.1126/science.abn8197
