# Supplementary material for: Constructing and interpreting a large-scale variant effect map for an ultrarare disease gene: Comprehensive prediction of the functional impact of PSAT1 genotypes
Source: PLoS Genet. 2023 Oct 9;19(10):e1010972. doi: 10.1371/journal.pgen.1010972 (PMC10561871; doi:10.1371/journal.pgen.1010972)
Supplement: S9 Fig — (DOCX) [file pgen.1010972.s009.docx]

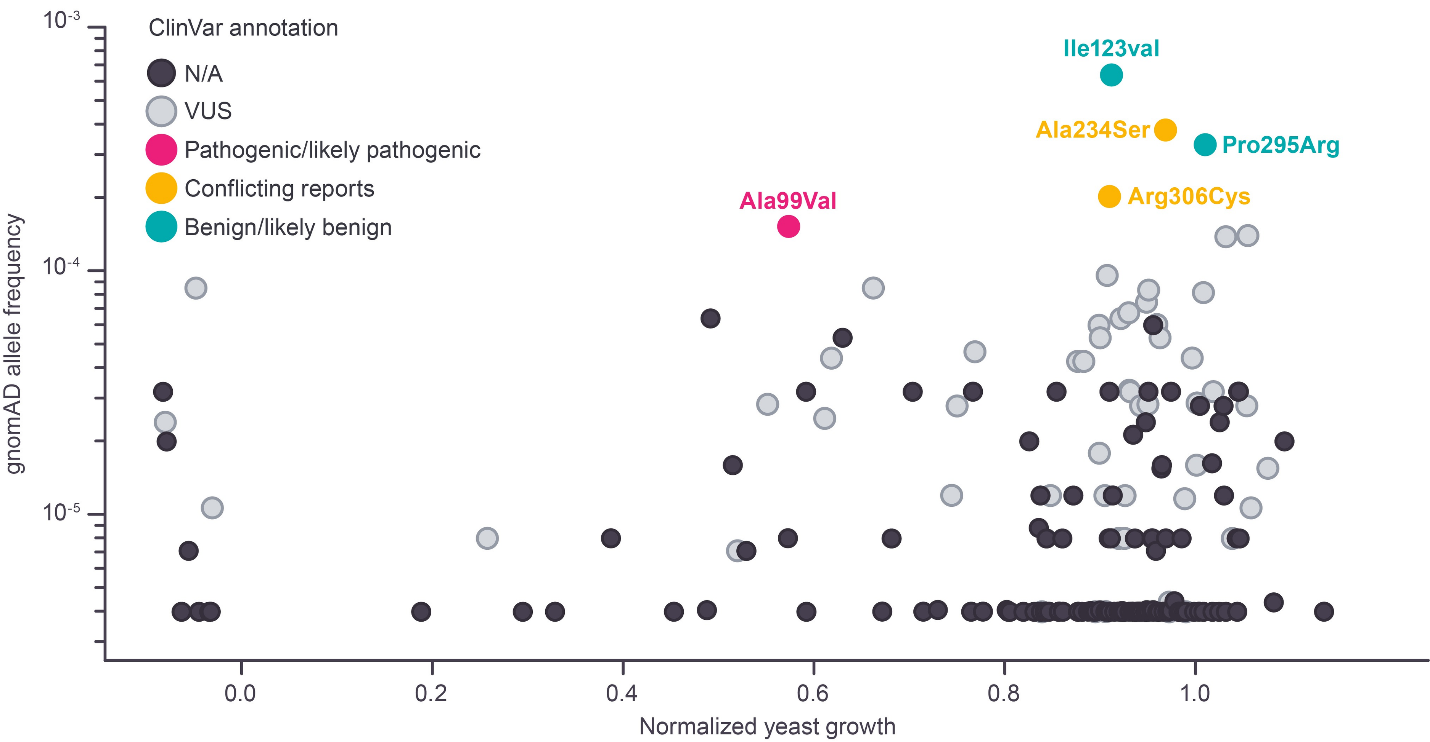


**S9 Fig**. **Allele frequency and yeast growth scores for PSAT1 missense variants found in gnomAD.** Scatterplot of normalized yeast growth scores and gnomAD (v2.1.1) [1] allele frequencies. Variants with ClinVar interpretations are colored according to the legend. Variants absent from ClinVar are colored black.

**Supplemental Reference**

1. Karczewski KJ, Francioli LC, Tiao G, Cummings BB, Alföldi J, Wang Q, et al. The mutational constraint spectrum quantified from variation in 141,456 humans. Nature. 2020;581: 434–443. doi:10.1038/s41586-020-2308-7
