# Supplementary material for: Constructing and interpreting a large-scale variant effect map for an ultrarare disease gene: Comprehensive prediction of the functional impact of PSAT1 genotypes
Source: PLoS Genet. 2023 Oct 9;19(10):e1010972. doi: 10.1371/journal.pgen.1010972 (PMC10561871; doi:10.1371/journal.pgen.1010972)
Supplement: S10 Fig — (DOCX) [file pgen.1010972.s010.docx]

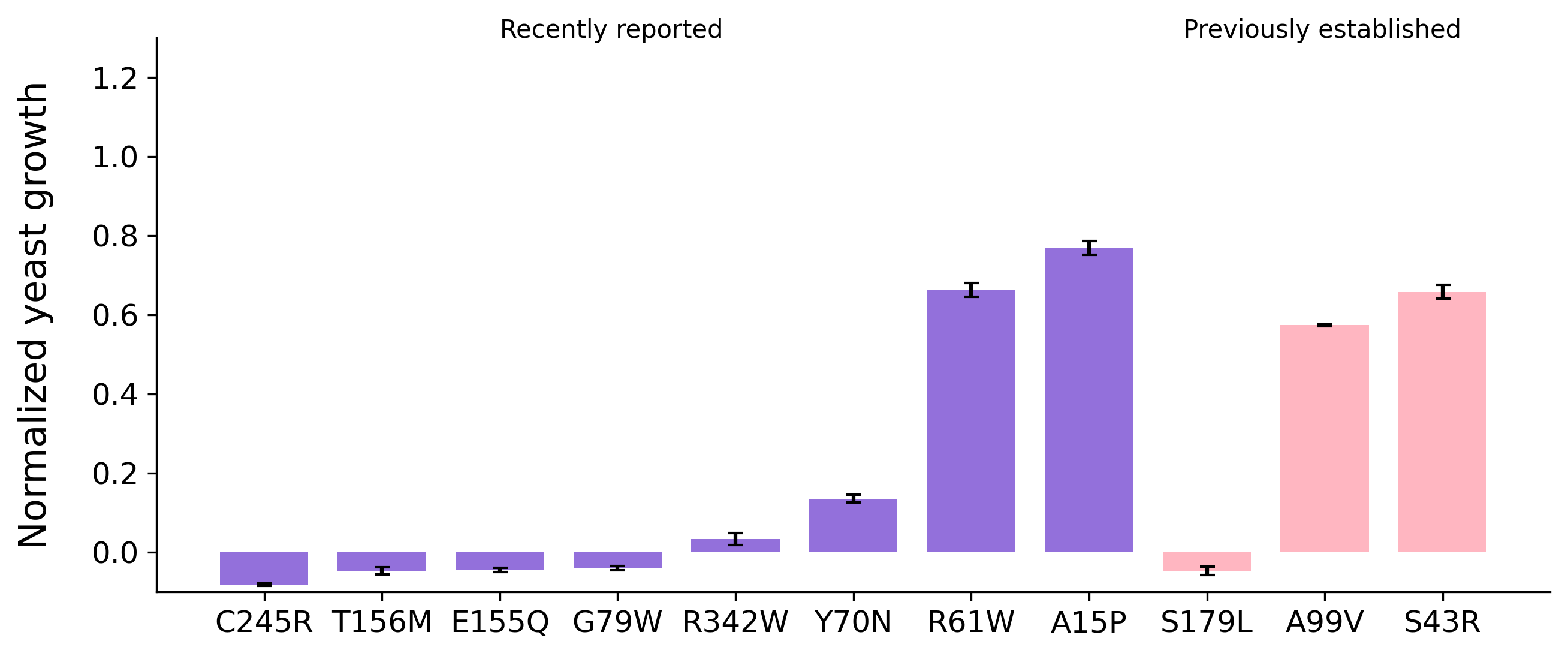


**S10 Fig**. **Haploid growth estimates of amino acid substitutions for missense alleles recently reported in the disease literature.** Bar plots of mean normalized haploid growth estimates for novel (purple) [1-4] patient alleles that result in missense substitutions. A set of previously established (pink) [5,6] missense alleles that have been observed in homozygous patient genotypes is also included for comparison. Errors bars indicate standard error.

**Supplemental References**

1. Abdelfattah F, Kariminejad A, Kahlert AK, Morrison PJ, Gumus E, Mathews KD, et al. Expanding the genotypic and phenotypic spectrum of severe serine biosynthesis disorders. Hum Mutat. 2020;41: 1615–1628. doi:10.1002/humu.24067

2. Debs S, Ferreira CR, Groden C, Kim HJ, King KA, King MC, et al. Adult diagnosis of congenital serine biosynthesis defect: A treatable cause of progressive neuropathy. Am J Med Genet A. 2021;185: 2102–2107. doi:10.1002/ajmg.a.62245

3. Shen Y, Peng Y, Huang P, Zheng Y, Li S, Jiang K, et al. Juvenile-onset PSAT1-related neuropathy: A milder phenotype of serine deficiency disorder. Front Genet. 2022;13. doi:10.3389/fgene.2022.949038

4. Ni C, Cheng RH, Zhang J, Liang JY, Wei RQ, Li M, et al. Novel and recurrent PHGDH and PSAT1 mutations in Chinese patients with Neu-Laxova syndrome. European Journal of Dermatology. 2019;29: 641–646. doi:10.1684/ejd.2019.3673

5. Acuna-Hidalgo R, Schanze D, Kariminejad A, Nordgren A, Kariminejad MH, Conner P, et al. Neu-laxova syndrome is a heterogeneous metabolic disorder caused by defects in enzymes of the l-serine biosynthesis pathway. Am J Hum Genet. 2014;95: 285–293. doi:10.1016/j.ajhg.2014.07.012

6. Brassier A, Valayannopoulos V, Bahi-Buisson N, Wiame E, Hubert L, Boddaert N, et al. Two new cases of serine deficiency disorders treated with l-serine. European Journal of Paediatric Neurology. 2016;20: 53–60. doi:10.1016/j.ejpn.2015.10.007
