# Supplementary material for: Constructing and interpreting a large-scale variant effect map for an ultrarare disease gene: Comprehensive prediction of the functional impact of PSAT1 genotypes
Source: PLoS Genet. 2023 Oct 9;19(10):e1010972. doi: 10.1371/journal.pgen.1010972 (PMC10561871; doi:10.1371/journal.pgen.1010972)
Supplement: S11 Fig — (DOCX) [file pgen.1010972.s011.docx]

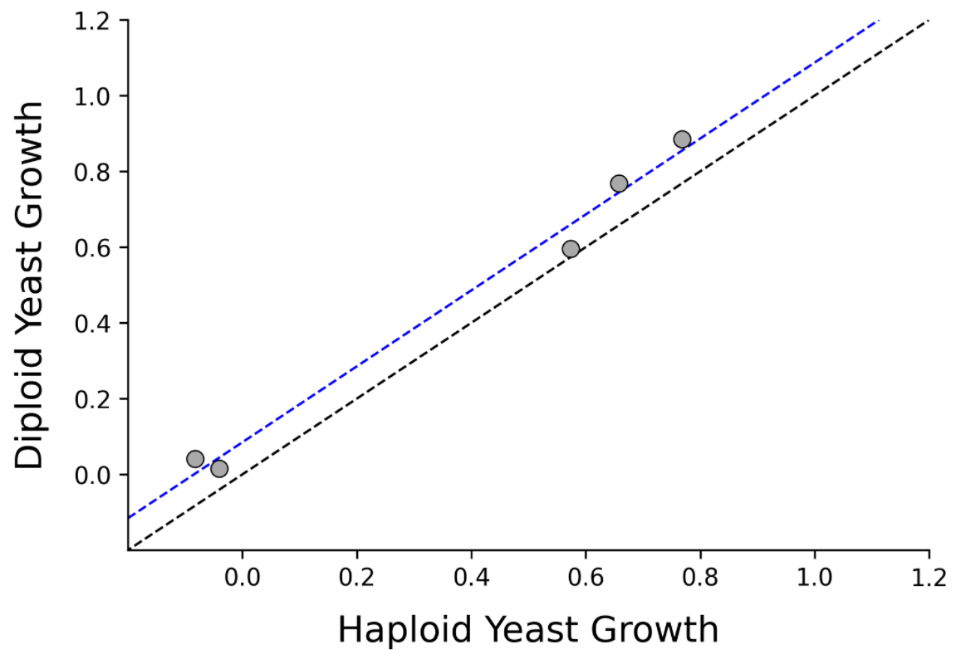


**S11 Fig**. **Haploid versus diploid yeast growth scores for alleles that are found in homozygous patient genotypes.** Scatter plot of normalized haploid and diploid growth scores. Haploid scores were scaled relative to wild type *yPSAT1* (normalized growth=1) and null (normalized growth=0). Diploid scores were scaled relative to homozygous wild type *yPSAT1 / yPSAT1* (normalized growth=1) and null / null (normalized growth=0). The black dotted line represents a 1:1 correspondence between the haploid and diploid scores. The blue dotted line indicated the observed correlation (R^2^=0.989) with slope of 1.0 and intercept of 0.09.
