# Supplementary material for: Constructing and interpreting a large-scale variant effect map for an ultrarare disease gene: Comprehensive prediction of the functional impact of PSAT1 genotypes
Source: PLoS Genet. 2023 Oct 9;19(10):e1010972. doi: 10.1371/journal.pgen.1010972 (PMC10561871; doi:10.1371/journal.pgen.1010972)
Supplement: S12 Fig — (DOCX) [file pgen.1010972.s012.docx]

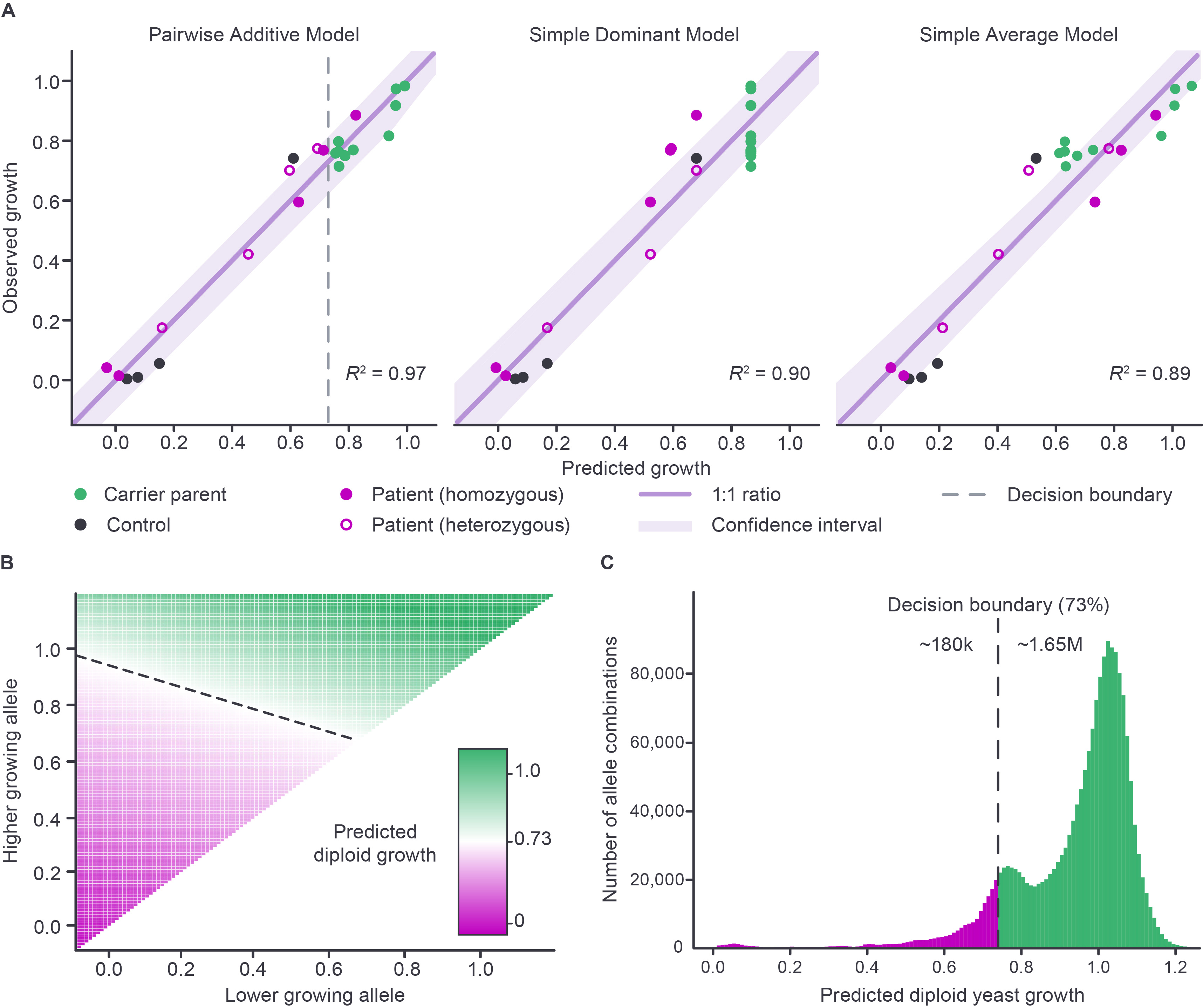


**S12 Fig. Comparing models for predicting diploid yeast growth representing biallelic combinations.** Observed versus fitted values shown for three linear regression models (pairwise additive, simple dominant, and simple average) predicting diploid growth (allele pairs) as a function of haploid growth (single alleles). Each circle represents a unique biallelic combination. Patient, carrier, and control strains are labeled as indicated. A diagonal line of perfect correspondence (1:1 ratio) and the coefficient of determination (R²) for each model are included for ease of comparison. The decision boundary for predicted diploid growth (73%) as a binary classifier for identifying patient genotypes modeled in our diploid assay is shown as a vertical dotted grey line. Confidence intervals (95%) for each model are shaded in purple.
