# Supplementary material for: Constructing and interpreting a large-scale variant effect map for an ultrarare disease gene: Comprehensive prediction of the functional impact of PSAT1 genotypes
Source: PLoS Genet. 2023 Oct 9;19(10):e1010972. doi: 10.1371/journal.pgen.1010972 (PMC10561871; doi:10.1371/journal.pgen.1010972)
Supplement: S13 Fig — (DOCX) [file pgen.1010972.s013.docx]

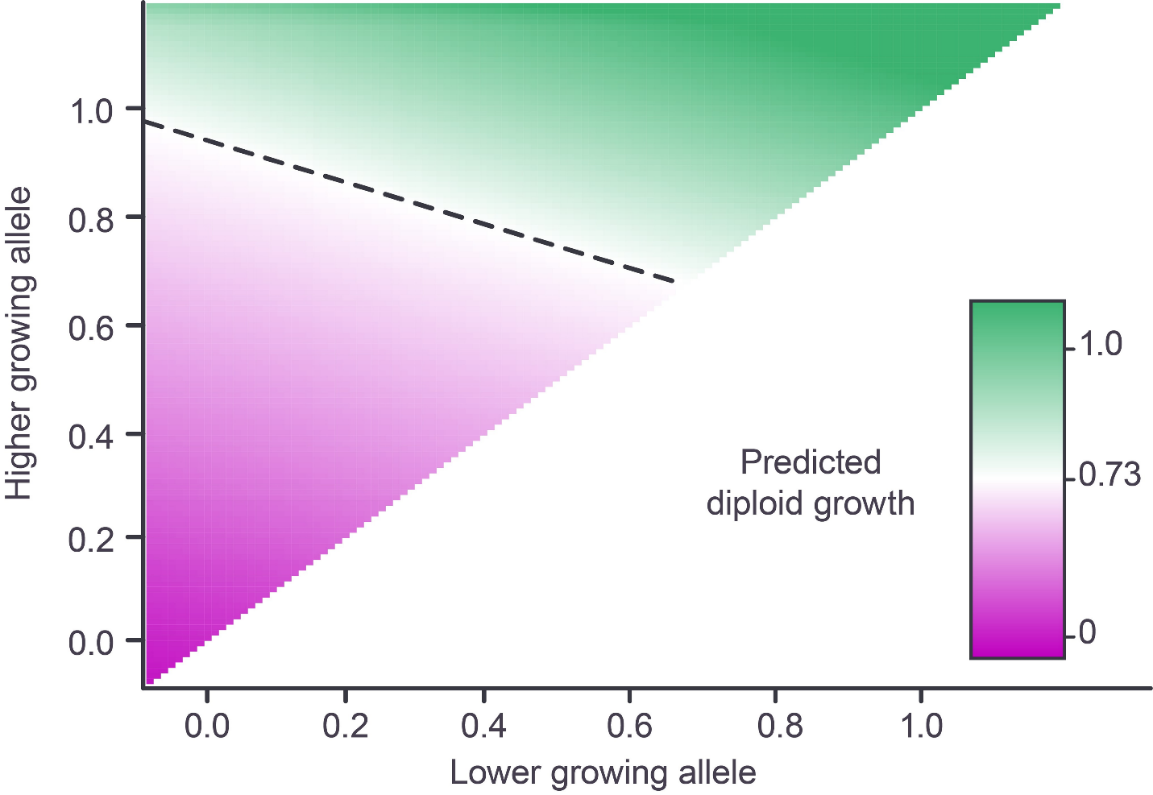


**S13 Fig**. **Heatmap of the predicted diploid growth value for all possible pairwise combinations of haploid allele measurements.** Distribution of predicted diploid growth scores from all possible pairwise combinations of haploid growth scores generated by the pairwise additive model. Predicted diploid growth is colored by a divergent coloring scheme, where values greater than the decision boundary (73% predicted diploid growth) are colored in shades of green and values lower as shades of magenta. Combinations of higher and lower growing allele scores that result in a predicted score at the boundary value are also marked by a dashed diagonal black line.
