# Supplementary material for: Constructing and interpreting a large-scale variant effect map for an ultrarare disease gene: Comprehensive prediction of the functional impact of PSAT1 genotypes
Source: PLoS Genet. 2023 Oct 9;19(10):e1010972. doi: 10.1371/journal.pgen.1010972 (PMC10561871; doi:10.1371/journal.pgen.1010972)
Supplement: S1 Text — (DOCX) [file pgen.1010972.s014.docx]

**Supplemental Methods**

**Determining the PSAT Variant Encoded by Each Isolate Using MinIon Sequencing**

A pooled approach utilizing Oxford Nanopore MinIon sequencing was used to identify the sequence variant present in each *YPSAT1* transformant. First, the 96-well variant plates (1 transformant per well) were collapsed in sets of 12, so that well A1 on each collapsed plate combined the variants in well A1 in each of 12 variant plates. Variant plates were ordered so that each of the 12 target codons present in each collapsed well were unique (S8-S13 Tables). Therefore, for each collapsed well, we know the 12 target codons present and we also know the set of potential variant sequences introduced at each codon by Twist Biosciences. By sequencing pooled DNA in each well of each collapsed plate and identifying the most common variant sequence at each target codon (among the set of potential variants), we can determine the identity of the variant in each well of the variant plates, i.e. in each transformant (S8-S13 Tables).

After collapsing the plates, fragments encompassing the *SER1* promoter, *yPSAT1* ORF, and *SER1* terminator were amplified from the pooled genomic DNA in each collapsed well using 16 or 18 cycles of PCR with dual barcoded primers (identifying each well). ONP adapters were added on via ligation. Sequencing was performed on an Oxford Nanopore MinION Mk1B with a Flongle adaptor and flowcell with 1 collapsed plate per run.

The reads from each sequencing run were demultiplexed to the pool level using MiniBar [1] with the parameters “-l 150 -F -S -M 1 -p .75”. Then, for each pool (collapsed well), reads were aligned to the *yPSAT1* reference sequence in R (R Core Team (2022). R: A language and environment for statistical computing. R Foundation for Statistical Computing, Vienna, Austria. URL https://www.R-project.org/) using the pairwiseAlignment function of the Biostrings package (Pagès H, Aboyoun P, Gentleman R, DebRoy S (2022). Biostrings: Efficient manipulation of biological strings. R package version 2.64.1, https://bioconductor.org/packages/Biostrings) with parameters gapOpening=10 and gapExtension=2. For each read, alignments were carried out in both orientations and the highest scoring alignment was kept. After alignment, at each target codon in the pool, the most frequent variant codon was identified (candidate variant) among the set of potential variant sequences introduced at that codon by Twist Biosciences. The second most frequent variant was also identified. Because we know which unique codon was targeted in each of the 12 transformant strains making up each pool, this gives us a candidate variant for each transformant.

The observed frequency of each candidate variant codon was then compared to the frequency expected under a variant-specific error model. To generate these models, for each pool we recorded the observed frequency of all possible Twist variants at codons NOT among the target set of 12 target codons in that pool. As these variants can only have been produced by sequencing errors, this gives us an error frequency estimate for each potential variant codon. On this basis, provisional variant calls were accepted if their observed frequency was >3.3 times their error frequency. In addition, candidates were rejected when the second most frequent variant was both enriched (>=10-fold) relative to its error frequency and was observed at >30% of the frequency of the candidate variant. Finally, candidates were rejected if they were supported less than 15 reads, or if any missense or nonsense secondary mutations were present in the *YPSAT1* sequence. The script carrying out the MinIon sequence processing steps for each pool (collapsed well) is provided as S1 Method. At the end of this process, for each transformant, we reported either a high confidence call for the variant present in that transformant, or an NA call resulting in that transformant being removed from analysis.

**Supplemental Reference**

1. Krehenwinkel H, Pomerantz A, Henderson JB, Kennedy SR, Lim JY, Swamy V, et al. Nanopore sequencing of long ribosomal DNA amplicons enables portable and simple biodiversity assessments with high phylogenetic resolution across broad taxonomic scale. Gigascience. 2019;8. doi:10.1093/gigascience/giz006
